# Aging disrupts blood–brain and blood-spinal cord barrier homeostasis, but does not increase paracellular permeability

**DOI:** 10.1007/s11357-024-01404-9

**Published:** 2024-10-30

**Authors:** Mitchell J. Cummins, Ethan T. Cresswell, Renee J. Bevege, Doug W. Smith

**Affiliations:** 1 Neurobiology of Aging and Dementia Laboratory, School of Biomedical Sciences and Pharmacy, College of Health, Medicine and Wellbeing, University of Newcastle, Callaghan, NSW Australia; 2 Brain Neuromodulation Research Program, Hunter Medical Research Institute, New Lambton, NSW Australia; 3 Present Address: School of Biotechnology and Biomolecular Sciences, UNSW Sydney, Sydney, NSW 2052 Australia

**Keywords:** Aging, Blood–brain barrier, RNA-Seq, Laser-capture, scRNA-seq, Proteomics

## Abstract

Blood-CNS barriers protect the CNS from circulating immune cells and damaging molecules. It is thought barrier integrity becomes disrupted with aging, contributing to impaired CNS function. Using genome-wide and targeted molecular approaches, we found aging affected expression of predominantly immune invasion and pericyte-related genes in CNS regions investigated, especially after middle age, with spinal cord being most impacted. We did not find significant perturbation of endothelial cell junction genes or proteins, nor were vascular density or pericyte coverage affected by aging. We evaluated barrier paracellular permeability using small molecular weight tracers, serum protein extravasation, CNS water content, and iron labelling measures. We found no evidence for age-related increased barrier permeability in any of these tests. We conclude that blood–brain (BBB) and blood-spinal cord barrier (BSCB) paracellular permeability does not increase with normal aging in mouse. Whilst expression changes were not associated with increased permeability, they may represent an age-related primed state whereby additional insults cause increased leakiness.

**Supplementary Information:**

The online version contains supplementary material available at 10.1007/s11357-024-01404-9.

## Introduction

Aging is associated with declining CNS function and compromised blood-CNS barrier integrity is thought to contribute to this [1–3]. Aging is proposed to impair endothelial cell (EC) tight junctions and/or pericyte cell (PC) signalling, which are critical to barrier ‘tightness’, resulting in neurotoxic and neuroinflammatory factors gaining access to CNS parenchyma.

Two of the blood-CNS barriers, the blood–brain barrier (BBB) and the blood-spinal cord barrier (BSCB) are formed by vascular ECs, PCs, astrocyte end-feet, and secreted basement membrane, with associated intercellular junctions and signalling [4, 5]. Tight junctions between ECs limit the ability of molecules and immune cells to move between the ECs via the paracellular pathway. The transcellular pathway, on the other hand, requires passage through ECs, conferring regulated entry of needed, and active or passive exclusion of unwanted molecules [4, 5]. Endothelial barrier permeability is influenced by PC signalling via the basement membrane and astrocytic end-feet that surround CNS vessels.

Whilst blood-CNS barrier permeability has been extensively studied, a consensus has not been established as to whether permeability increases with aging [6]. Human studies using the ratio of cerebrospinal fluid (CSF) to serum albumin as a measure of barrier permeability, generally report increases with age [7, 8]. However, a major limitation of this approach is lack of barrier specificity, as the measure is more an indicator of blood-CSF barrier and not BBB or BSCB function [9]. Human studies using high resolution, contrast-enhanced MRI, have yielded equivocal results [1, 10, 11]. Human post-mortem histological studies have found age-related increases in extravasated blood proteins in brain, which has been interpreted as indicative of BBB leakiness [3, 10].

In animal models, evidence for age-related changes in CNS barrier permeability is similarly mixed. Studies using exogenous tracers have reported no effects [3, 12, 13] or an increase in tracer extravasation with aging [10, 14–17]. Endogenous blood protein extravasation has also been investigated in animals. Again, studies report either no effects [18] or increases in leakiness to blood proteins with aging [10, 14, 19–21]. Another indicator of declining CNS barrier integrity is loss of pericyte coverage. Some studies have found up to 50% decline in pericyte coverage [20, 21] while others have found no age effect [14, 18]. The reasons for these discordant findings are not known but need to be if we are to understand all factors contributing to aging’s impacts on the blood-CNS barriers (for a comprehensive review on the contentious issue of normal aging and BBB structure and function see [6]).

To improve our understanding of the impact(s) of aging on blood-CNS barriers, we first carried out deep RNA-seq on cortex (CTX), and spinal cord (SC) from young and old age mice. This discovery-based approach was followed by more targeted analyses including additional CNS regions (hippocampus (HIP) and cerebellum (CB)), using an array of approaches spanning molecular and proteomics, through to functional, with a focus on assessing the paracellular pathway and endothelial cell junctions.

## Methods

A detailed description of procedures can be found in Online resource 1.

### Animals

Young (2–4 months) and old (> 24 months) C57BL/6 male mice were used for all studies. All animal work was undertaken in strict accordance with the University of Newcastle Animal Ethics Committee, and New South Wales and Australian animal research guidelines.

### Tissue dissection and cryosectioning

For molecular analyses, mice were perfused with phosphate buffered saline (PBS), brains and SCs were frozen in isopentane on dry ice. Cryosections were collected onto microscope slides. All reagents and procedures were RNase free and carried out on ice where appropriate.

For immunolabelling and staining analyses, mice were perfused with PBS, followed by 4% paraformaldehyde (PFA). Tissues were post-fixed, cryosectioned at 40 µm, and stored in PBS at 4 °C.

### Molecular analyses – gene expression

#### Bulk RNA sequencing

- Tissue preparation for bulk RNA sequencing
  RNA was extracted from cervical SC and frontal CTX. DNA-free, RNA samples were sent to the Australian Genome Research Facility for sequencing.
- Analysis of CNS barrier related gene expression by RNA sequencing
  RNA sequencing was carried out to generate lists of differentially expressed genes (DEGs) for analyses for enrichment of CNS barrier-related genes. Total RNA was prepared for sequencing using Illumina platforms and reads were aligned using STAR. Aligned files were assessed to determine DEGs using Cuffdiff. DEG lists for the CTX and SC were compiled using a FDR cut-off of < 0.05. DEG lists were analysed for Gene Ontology (GO) enrichment using the PANTHER Overrepresentation Test for biological process (https://geneontology.org/). DEG lists were compared with the CNS barrier-related gene lists in Supplementary Tables S1-4. For cell type-specific gene sets (see Supplementary Table S1) we curated the sets from multiple published databases (database details can be found in Online resource 1). We used the criterion a cell-type-specific gene had to be annotated in the same CNS cell type in at least 2 databases. Enrichment of CNS barrier-related gene sets in the DEG lists was determined using a hypergeometric overlap calculator: https://systems.crump.ucla.edu/hypergeometric/. *p*-values were corrected for multiple comparisons.

#### Age-related barrier gene expression across CNS regions by qPCR

All qPCR primer sequences for all experiments are available in Supplementary Table S5. Sample RNA integrity measured using the 3' to 5' ratio for all experiments is available in Supplementary Table S6.

To confirm RNA-seq outcomes and to add white matter (WM) regions to our analysis, qPCR analyses were carried out on grey (GM) and WM CNS regions. Brains and SCs were cryosectioned at 100 µm thickness and CTX, corpus callosum (CC), hippocampus (HIP), CB, cervical spinal cord GM (SCGM), cervical spinal cord WM (SCWM), dissected out, RNA extracted and reverse transcribed.

Genes for qPCR analysis were selected based on importance to CNS barrier function, and on our RNA-seq results. qPCR reactions were run on Applied Biosystems 7500 or QuantStudio 6 Pro devices.

Relative expression differences between age groups were determined using the comparative Ct method. Statistical significance was determined using one-tailed Wilcoxon Mann-Whiney U Exact test. A *p*-value of *p* < 0.05 was applied. All *p*-values were adjusted for multiple comparisons using the sequential Holm-Bonferroni procedure.

Based on RNA-seq and qPCR, the SC was the most affected region and was the focus of time course analysis. Mice across five age groups (2.5 months, 4 months, 8 months, 14 months, and 26 months) were used. RNA was extracted and reverse transcribed from whole cervical spinal cords. qPCR reactions were run as above. Differences between age groups were determined using the Kruskal–Wallis Test followed by a post-hoc Steel–Dwass Test with the 2.5 months old group as the control, with *p* < 0.05 for significance.

#### CNS blood vessel-specific gene expression using laser microdissection and qPCR

Brains and SCs were cryosectioned at 10 µm. Sections were acetone fixed and incubated overnight in rabbit anti-collagen IV (ColIV) antibody in 2 M NaCl PBS for RNA protection [22]. After rinsing, sections were incubated with Alexa Fluor 594 conjugated donkey anti-rabbit antibody in 2 M NaCl PBS. Immediately prior to microdissection, slides were dehydrated and delipidated. Microdissection was carried out using a PALM MicroBeam system (Zeiss). Blood vessel profiles were collected from GM regions only. qPCR and data analyses were done as above.

### Single cell meta-analysis

Publicly available gene-count data and metadata from all studies was downloaded from the Gene Expression Omnibus (see Supplementary Table S7 for series identifiers). Data was analyzed in R/4.0.1 using Seurat v3.2.2. Young mice were 2–3 months old and old mice were 18–24 months old.

For each study, cells with fewer than 200, and greater than 5,000 detected genes were removed. The Seurat tutorial (https://satijalab.org/seurat/articles/integration_introduction.html) for dataset integration was followed. In brief, each dataset was normalized separately using NormalizeData, and features were selected using FindVariableFeatures “vst” selection method selecting 2,000 features. Features that were variable across datasets were selected using the SelectIntegrationFeatures. Anchors for integration were identified using FindIntegrationAnchors and datasets were integrated using IntegrateData. Data was scaled and centered using ScaleData.

Clustering and visualization using the RunUMAP, FindNeighbours, and FindClusters was performed with defaults except for FindClusters with 0.3 resolution. Cluster marker genes were identified using FindAllMarkers for positive markers with minimum logfc.threshold of 0.25. Young and old cells in each cluster for each study were counted using the subset function to select age group and study name, and table function to select cell cluster metadata for the cell subset. Clusters were identified by searching for cluster marker genes using 2 databases (https://www.brainrnaseq.org/ and http://mousebrain.org). Differential expression testing between young and old cells in each cluster was performed using FindMarkers with grouping by “Age” and subset by cell cluster, with a minimum logfc.threshold of 0.25.

### Protein analyses

The effects of aging on Claudin 5 (Cldn5) and Occludin (Ocln) protein expression were quantitatively assessed by Western blot. In brief, total protein extracts from SC and CTX of young and old mice (*n* = 4/5) were separated by SDS-PAGE, transferred to nitrocellulose membranes, and stained for total protein (Revert, LI-COR). Membranes were then blocked and incubated overnight with either Cldn5, Ocln, or Tubulin beta 3 (Tubb3) primary antibodies. Following fluorescent secondary antibody labelling, blots were scanned in the 700 and 800 nm channels on an Odyssey M imaging system (LI-COR) and images analysed using ImageStudio (LI-COR). Protein bands were normalised using both total protein and Tubb3 levels. Additionally, two proteomic databases were evaluated for the effects of aging on BBB-related protein changes [23, 24]. Detailed methods can be found in the Online resource 1.

### Blood vessel density and pericyte coverage

For blood vessel density, 4–6 fixed sections per animal, equidistant across each CNS region, were immunolabelled for ColIV. Sections were rinsed and incubated with AF594 fluorescent secondary antibody. Sections were washed, mounted in gelvatol, coverslipped, and imaged on a Nikon D-Eclipse C1 confocal microscope. Z-stacks were captured using a 40 × objective, and the area of ColIV labelling relative to total area quantified using ImageJ using maximum intensity projections. Differences between groups were assessed using a nested t-test (sample nested within the age group), with a *p* < 0.05 for significance of the age effect.

Pericyte coverage was determined for SC by co-immunolabelling for CD13 and ColIV. Sections (6/animal) were confocal imaged as above. Relative areas covered by ColIV and CD13 were quantified using ImageJ as for vessel density. Percentage area of pericyte coverage was calculated using: ((area of CD13 labelling / area of ColIV labelling) * 100). Differences between groups were assessed using a nested t-test, with a *p* < 0.05 for significance of the age effect.

### Barrier functional analyses

#### CNS water content

Brains and SCs were extracted, weighed to determine tissue wet weight, then dried at 85 °C for 7 days. Wet weight, dry weight, and percentage CNS water content ((wet-dry)/wet*100) were calculated.

#### Exogenous CNS barrier tracers

- Sodium Fluorescein (MW 376.3 Da)
  Mice were injected i.p. with 200 µl 6% NaFl and euthanized after 15 min. Blood was collected, animals perfused with PBS, and samples from frontal CTX, CC, HIP, CB, and cervical SC, were weighed, frozen on dry ice, and stored at -80 °C. Serum was aspirated from blood cells and stored at -80 °C.
  Tissue proteins were precipitated with tri-chloroacetic acid in preparation for NaFl fluorometry on a microplate reader. Sample fluorescence was normalised to sample serum fluorescence and sample tissue weight.
- Fluorescein Dextran (MW 3 kDa)
  Mice were injected i.p. with 400µL 5 mg/mL 3 kDa lysine fixable fluorescein dextran and euthanized after 10 min. Tissues were drop-fixed overnight in 4% PFA. Brain and spinal cord sections were visualized on an Olympus BX51 epi-fluorescent microscope. The number of extravascular leakages was counted and normalised to the number of sections assessed.

#### Endogenous indicators of CNS barrier function

- Serum Albumin (MW approx. 69 kDa)
  Brain and spinal cord sections were co-immunolabelled for mouse serum albumin and CD31. Sections were assessed on an Olympus BX51 epi-fluorescent microscope and the number of areas demonstrating perivascular serum albumin were counted and normalised to the number of sections counted.
- IgG (MW approx. 150 kDa)
  Brain and spinal cord sections were prepared for co-immunolabelling for ColIV and IgG, and sections were assessed as described for serum albumin labelling.
- Iron labelling for microhaemorrhages
  Brain and spinal cord sections were stained for iron using potassium ferrocyanide. Dehydrated and delipidated sections were mounted in ultramount and assessed on an Olympus BX51 microscope under bright field illumination. The number of microhaemorrhages were counted and normalised as described for serum albumin labelling.
  Group differences for all functional analyses were assessed using a one-tailed Wilcoxon Mann–Whitney U Exact test, with a corrected *p*-value of < 0.05.

## Results

### Expression of immune invasion, pericyte, and extracellular matrix genes is perturbed by aging, but endothelial junction gene expression remains stable

#### Aging blood-CNS-barrier transcriptomes indicate BBB/BSCB dysfunction

We deep sequenced RNA from CTX and SC of young and old mouse CNS. The sample read depth for the 100 bp, paired-end reads was 81.9 ± 2.3 (average ± SEM) million. The complete gene expression datasets for both CNS regions and DEGs from aging comparisons (q < 0.05) and GO enrichment analyses are in Online resource 2.

**Fig. 1:**
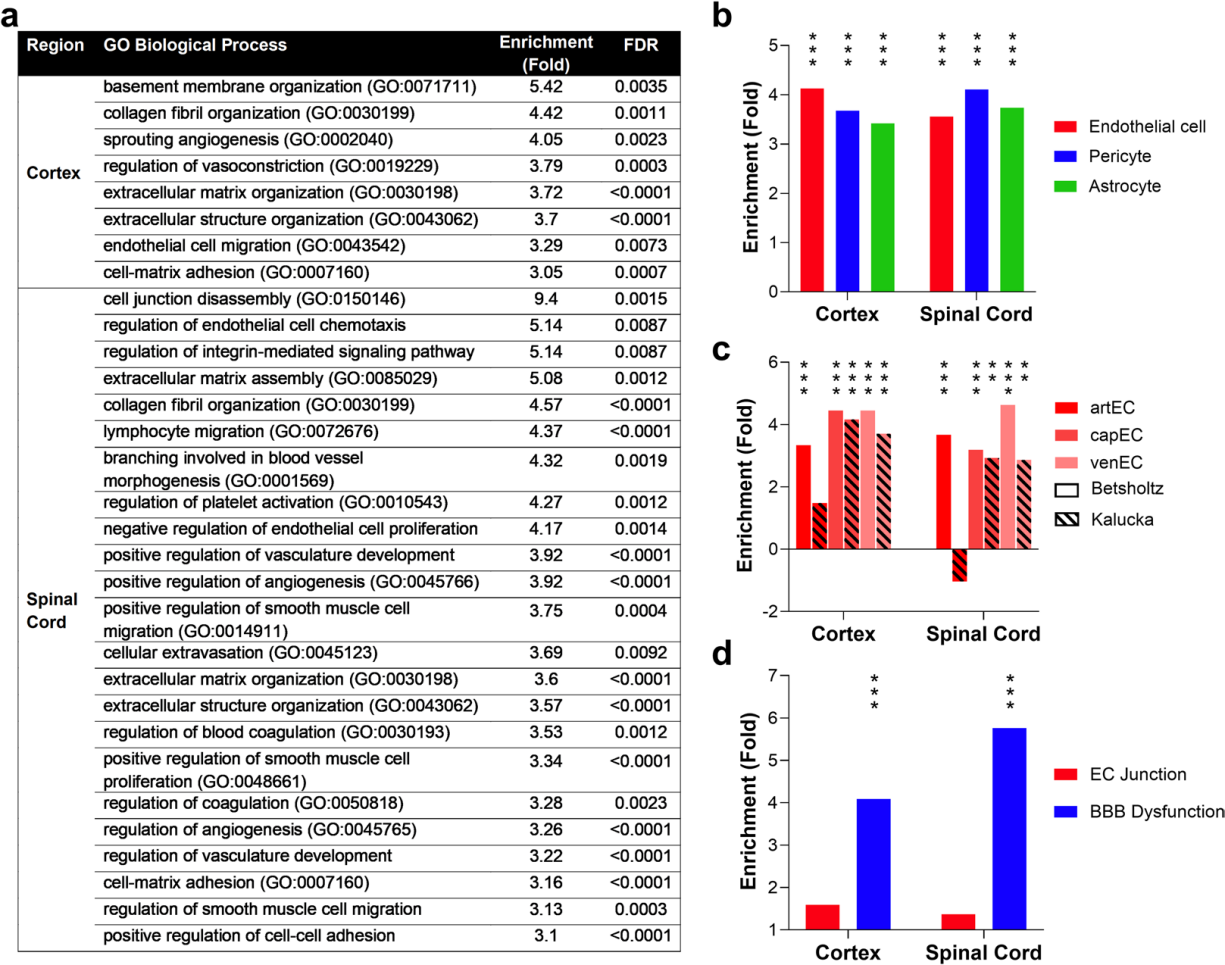
RNAseq of CTX and SC indicates barrier transcriptomes are perturbed by aging. **a** Barrier-related GO enriched in CTX and SC DEG sets with aging. GO with enrichment > 3 and FDR < 0.01 are shown. **b** Enrichments plotted for endothelial- (EC), pericyte-, and astrocyte-cell-type-specific genes in the DEG sets for CTX and SC, using cell-type-specific gene sets as described in the text. **c** Enrichments plotted for ECs located at different parts of the cerebrovasculature tree. **d** Enrichments plotted for EC junction genes and BBB dysfunction module genes. See Methods for details on enrichment analyses. **b-d** **p < 0.01 ***p < 0.001

There were 173 enriched GOs in CTX and 761 in SC (enrichment score > 2, FDR < 0.01). Of these, 22 CTX and 36 SC enriched GOs were related to blood vessels or cell junctions (Supplementary Table S8), suggesting components of the BBB/BSCB are affected by aging. Figure 1a shows a subset of barrier-related enriched GOs (> threefold enrichment). To gain a more detailed understanding of which CNS barrier components may be affected by aging, DEGs were compared to experimentally-determined gene sets. We used barrier cell-type-enriched gene sets derived from improved cell-type-specific approaches and meta-analyses. To reduce variability in cell-type-specific gene sets, we used an ‘at least in two gene sets’ criterion for gene inclusion (see Methods). This compilation approach yielded 287 endothelial-, 136 pericyte-, and 141 astrocyte-cell-specific genes for enrichment analyses (cell-specific-gene sets can be found in Supplementary Table S1). Figure 1b shows the results of cell-type enrichment analyses, which indicate all barrier cell types are impacted by aging. Additionally, we made use of recently characterised cerebrovasculature tree-region-dependent EC molecular profiles [25, 26] to determine if aging impacts ECs in all regions of the tree similarly (Fig. 1c, Supplementary Table S2). Interestingly, venule ECs appear more impacted by aging in the SC compared to capillary and arteriolar ECs, although this pattern was not replicated with the [26] data. Cerebrovascular tree regions are responsible for separate, albeit related, functions. In general, arterioles control blood flow and pressure, capillaries mediate entry and exit of molecules from the CNS parenchyma and modulate local blood flow in response to stimuli, and venules mediate immune cell invasion [5, 27–29]. Whether the apparent greater impact of aging on SC venule ECs has functional implications requires further investigation.

Junctions between ECs are critical to barrier integrity. We used a 70-gene, brain EC junction gene set [30], to assess impact of aging on EC junctions (Supplementary Table S3). There was no significant EC junction gene set enrichment in CTX or SC, indicating no or minimal aging effects (Fig. 1d, *p* > 0.05), at least not at a gene set level (see below for individual genes). Munji et al. [30] also established a barrier dysfunction gene set comprising 136 genes upregulated in disease or injury (Supplementary Table S4). We found significant enrichment for BBB dysfunction genes with aging (Fig. 1d), with 22% and 36% being differentially expressed in CTX and SC, respectively. Note, only one gene is common to both EC junction and dysfunction gene sets, therefore these enrichment analyses are assessing different aspects of barrier function. All hypergeometric test results are in Supplementary Table S9. Overall, our RNA-seq and enrichment analyses indicate blood-CNS barriers become perturbed with age, with SC being impacted to a greater extent than CTX. While all barrier cell types are affected, EC junction-specific genes are not significantly impacted.

#### Barrier expression changes are most prevalent in the aging SC and occur post-middle age

**Fig. 2:**
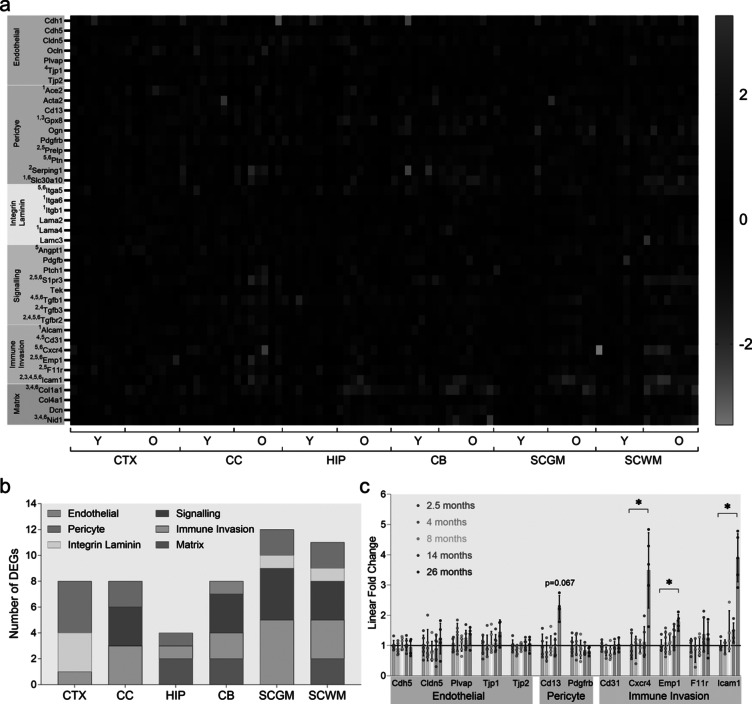
BSCB is more impacted by aging than BBB. **a** Heatmap of barrier gene expression. Individual sample expression is relative to average expression of young in matching CNS region. Scale bar is log_2_ FC. Superscripted numbers with gene names indicate region(s) reaching statistical significance: CTX^1^ CC^2^ HIP^3^ CB^4^ SCGM^5^ SCWM^6^. **b** Number of significantly differentially expressed genes (DEGs) of each functional group in each CNS region. **c** Relative expression of selected barrier genes across mouse adult lifespan (2.5, 4, 8, 14, 26 months) in SC. Expression is relative to 2.5-month group. Dots indicate individual animals. Bars indicate mean. Error bars ± SD. *p < 0.05 corrected. CTX – cortex; CC – corpus callosum; HIP – hippocampus; CB – cerebellum; SC – spinal cord; GM – grey matter; WM – white matter

To gain a more complete understanding of regional heterogeneity in barrier aging, we investigated age-related gene expression changes in 41 barrier-related genes in the CTX, CC, HIP, CB, SCGM and SCWM, from the same animals using qPCR. Genes were functionally grouped, as follows: ECs (7 genes), PCs (10), immune cell invasion (6), extracellular matrix (ECM) (4), integrin-laminin binding (6), and genes involved in signalling between barrier cell types (8). Gene level expression results are presented in Fig. 2a. Supplementary Table S10 contains group fold changes, and *p*-values for all comparisons.

Based on number of significant DEGs and consistent with the RNAseq analyses, SC was the most affected region, with differential expression of 12/11 (GM/WM) of 41 genes. CTX, CC, and CB, were the next most impacted, with expression changes in 8 genes each. Only 4 genes were affected in HIP, a region generally considered to be of greater susceptibility to aging processes. No single gene was differentially expressed in all 6 regions, although immune signalling Icam1 was a DEG in 5/6 regions. Aging affected genes from all six functional groups, but in a region-specific way. Immune cell invasion was the only functional group perturbed in all 6 regions, although DEGs in this group were not strictly the same. PC genes were the next most widely affected, with differential expression in all regions except CB. Of the signalling genes investigated, the Tgf-related pathway was most impacted, with the receptor Tgfbr2 being upregulated in 4 regions. Figure 2b. summarises the impact of aging on functional groups across CNS regions investigated.

We also assessed the age-progression of barrier expression changes. As SC was the most aging-affected CNS region, we investigated BSCB expression changes across the mouse adult lifespan. Only 3 of the 12 genes assessed were differentially expressed. All were immune cell invasion related (Cxcr4, Emp1, Icam1), overexpressed, and significance was not reached until after 14 months (Fig. 2c). The apparent increase in Cd13 expression did not survive correction for multiple comparisons. The complete dataset is available in Supplementary Table S11. These results indicate impacts of aging become evident post-midlife.

#### EC-PC-specific gene expression analysis through microdissection

**Fig. 3:**
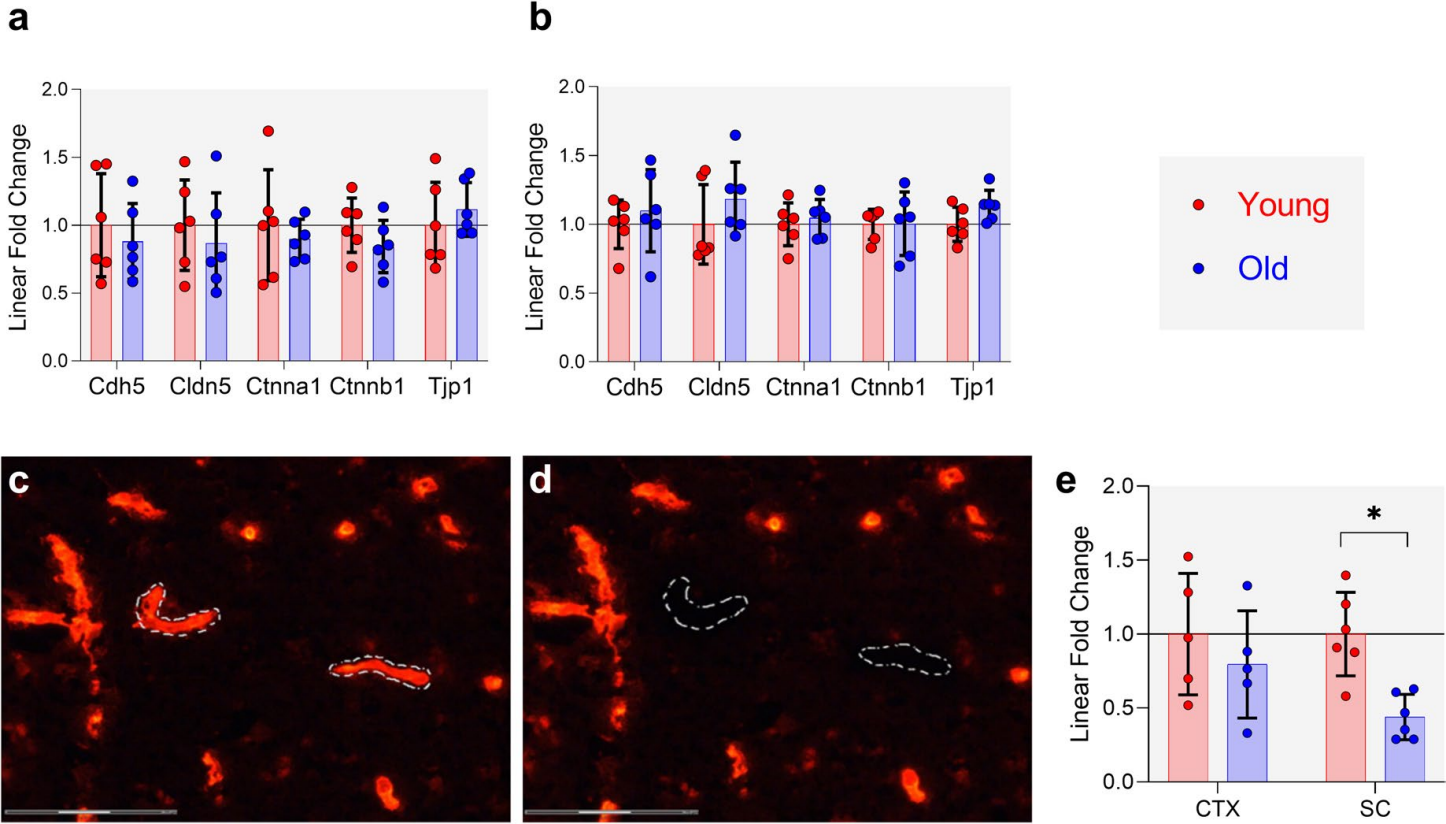
Laser microdissection of CNS microvessels. Relative expression of barrier junction genes in **a** CTX and **b** SCGM laser microdissected blood vessels. Col-IV-immunolabelled 10 µm SC section **c** before and **d** after laser microdissection of microvessels, 40 × objective. Scale bars are 75 µm. **e** Relative expression of F11r in CTX and SC blood vessels. Bars represent group fold change relative to young, points represent individual animals relative to young group average, error bars are SD for all graphs. *n* = 5–6/group.* one-tailed Wilcoxon Mann-Whiney U Exact test corrected *p*-value < 0.05. CTX – cortex; SCGM – spinal cord grey matter

To obtain a more direct measure of barrier gene expression, we extracted RNA from microdissected microvessels for qPCR analysis (Fig. 3). CNS blood vessels were immunolabelled for Col-IV, a collagen secreted by both EC and PCs. Microdissection of Col-IV-positive profiles therefore samples these two cell types, but limited sample tissue necessitated a more targeted list of genes to investigate. EC junction-related genes Cdh5, Cldn5, Ctnna1, Ctnnb1 and Tjp1 were not differentially expressed in CTX or SC GM microvessels (Fig. 3a-b). Figure 3c-d show examples of pre- and post-laser microdissection of immunolabelled Col-IV profiles and demonstrate the high spatial and cellular resolution with this approach. The tight junction and immune related F11r (junction adhesion molecule A, Jam-A) decreased in SC GM microvessels only (Fig. 3e). The complete microvessel-targeted gene expression dataset is available in Supplementary Table S12.

### Meta-analysis of single-cell-RNA-seq studies

**Fig. 4:**
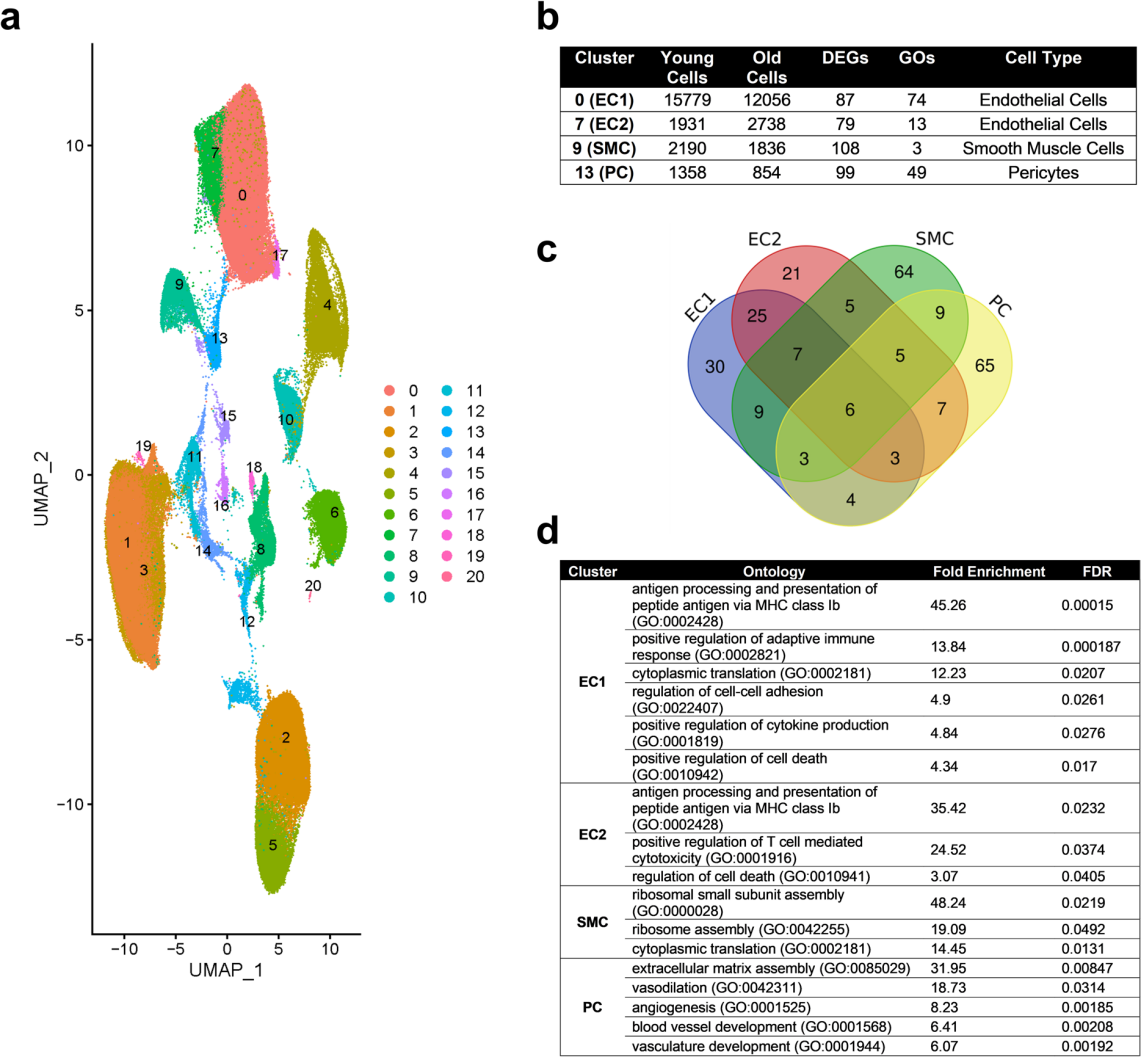
Aging brain single cell meta-analysis. **a** UMAP of aging brain cells. Putative cell type clusters: 0—ECs; 1—microglia; 2—astrocytes; 3—microglia; 4—oligodendrocytes; 5—astrocytes; 6—oligodendrocyte precursor cells; 7—ECs; 8—neurons; 9—smooth muscle cells; 10—choroid plexus epithelial cells; 11—perivascular macrophages; 12—ependymal cells; 13—PC; 14—perivascular macrophages; 15—vascular leptomeningeal cells; 16—vascular leptomeningeal cells (ABC); 17—olfactory ensheathing cells; 18—neurons; 19—proliferating; 20—oligodendrocytes. **b** Number of cells in EC and mural cell clusters. **c** Venn diagram of EC and mural cell clusters age-related DEGs **d** Selected enriched GOs of DEGs in cluster 0 (EC1), cluster 7 (EC2), 9 (SMC), and 13 (PC). EC – endothelial cell; SMC – smooth muscle cell; PC – pericyte

We complemented our molecular analyses with a meta-analysis of mouse whole brain single-cell datasets from five studies (Supplementary Table S7). Combining data from 152,696 cells to improve statistical power. Gene expression data from 80,929 and 71,767 brain cells from young and old mice, respectively, were analysed using Seurat [31]. Twenty-one cell clusters were identified (Supplementary Table S13), with 2 overlapping clusters (0 and 7) of ECs (Fig. 4a-b), and two clusters (9 and 13) of vascular mural cells. Two main subclasses of mural cell populations, PCs and smooth muscle cells (SMCs), were also reported in a recent analysis of the murine brain cerebrovascular tree [25]. As PCs are comparatively rare CNS cells, past differential gene expression comparisons may have been underpowered. The present PC meta-analysis reduces this concern. The DEGs within each cluster are listed in Online resource 3.

Mural cell (PCs and SMCs) clusters contained a greater number of DEGs (207) than EC clusters (166). Venn diagram analysis (Fig. 4c) showed little DEG overlap between PCs and SMCs (23 genes), whereas there was substantial overlap between the two EC clusters (41 genes). Relatively few DEGs overlapped different cell types and only 6 DEGs were common to all four cell clusters.

The EC clusters were screened for expression changes in genes that impact barrier function: the adherens (VE-cad, Pecam1, Nectin2, Afdn), tight (Tjp1-3, Ocln, Cldn5), and gap (Gja-e) junctions. Only Gja1 (a.k.a connexin 43) expression was affected, being lower with aging (Online resource 3), consistent with recent work [32]. Septin 2 (Sept2) is thought to organise EC junctional proteins [33] and was upregulated in both EC clusters (Online resource 3). Interestingly, expression of junctional cadherin 5 associated (Jcad), a risk allele for coronary artery disease, was significantly increased in EC cluster 7. Jcad encodes a protein that disrupts EC function and promotes atherosclerosis [34, 35].

For mural cells we focussed primarily on mural cell-EC signalling that is critical for barrier formation and maintenance [36]. There were no significant expression changes in Pdgfb, Tgfb, Angpt/Tek, nor Ncad signalling genes (Online resource 3). However, there were a number of basement membrane genes that were age-affected including Col1a2, Col4a2, Lamb1, Spock2, Tnxb, Dmp1, Vtn, Nupr1, and Ogn. The collagens, Lamb1, Tnxb and Dmp1 had reduced expression while Spock2, Vtn, Nupr1, and Ogn were increased with aging.

GO analyses revealed that EC and SMC clusters were enriched for a small variety of GOs. Example enriched GOs are shown in the table in Fig. 4d for all clusters. Many EC cluster enriched GOs involve immune functions (40 of 87 enriched GOs for both EC clusters, FDR *p* < 0.05), for example antigen processing and presentation, and adaptive immune system regulation involving T cells. Other EC enriched GOs involved cell death, protein processes, cellular responses to various stimuli, wound healing, and haemostasis. Consistent with there being separate EC clusters, cluster 0 had a greater number of and more diverse enriched GOs than EC cluster 7. While mural cell clusters had more DEGs than EC clusters, the number of enriched GOs was smaller (52, FDR *p* < 0.05), indicating fewer biological process were perturbed by aging. Indeed, SMCs only had 3 significantly enriched GOs, all involving protein translation. PCs, however, were enriched for a diverse array of GOs including many related to blood vessel development and maintenance (14 of 49), the extracellular matrix (3/49), development, proliferation, and differentiation processes (8/49), synaptic signalling by gas (5/49), response to oxygen levels (1/49), and other general metabolic and cellular processes (see Supplemental Table S14 for the complete GO enrichments).

### Protein analyses

**Fig. 5:**
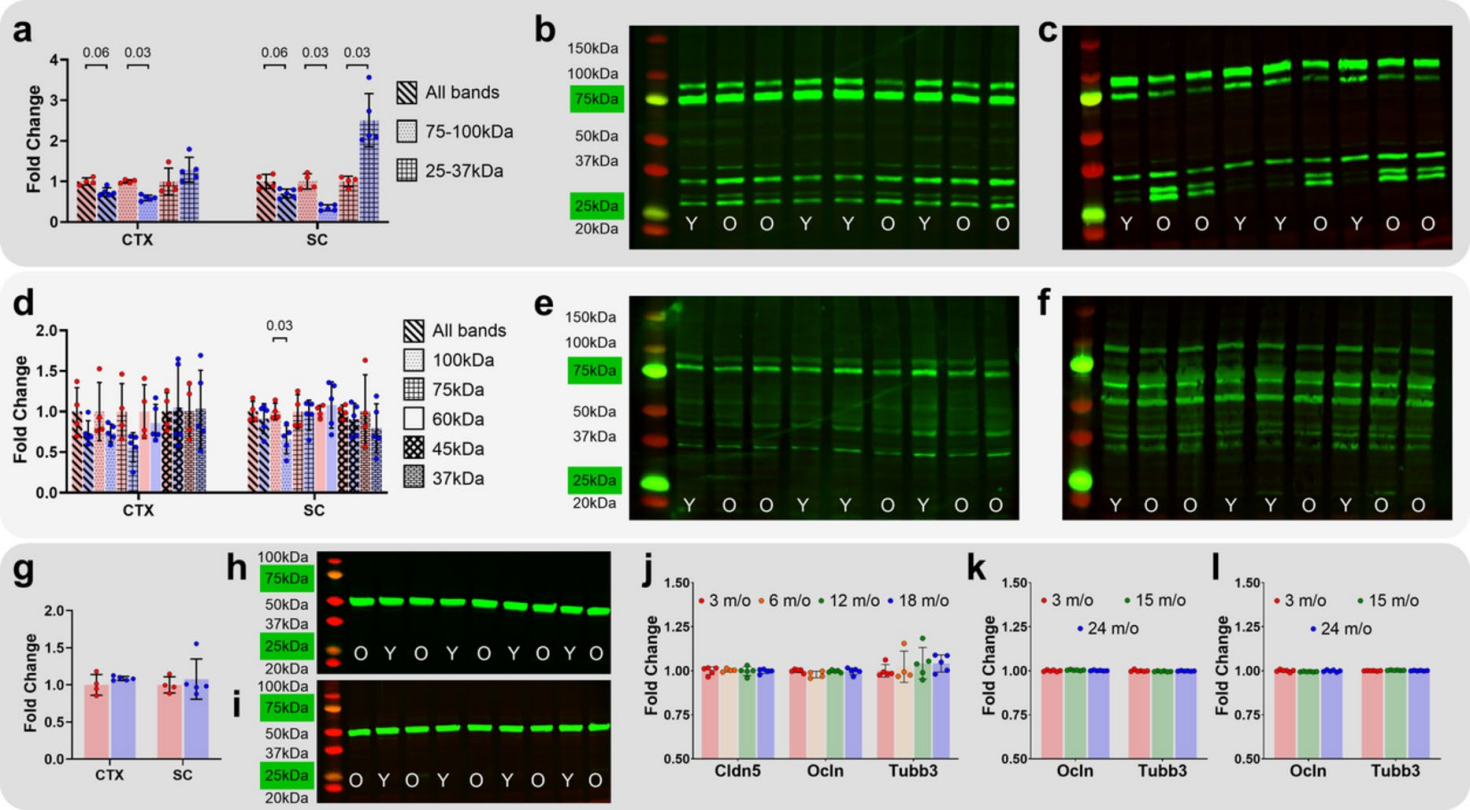
Effects of aging on blood-CNS barrier proteins. **a** Quantification of claudin-5 protein expression in CTX and SC of young (red columns) and old (blue columns) mice. Columns represent different MW bands of the protein. Western blots from which expression was quantified are shown for **b** CTX and **c** SC. **d** Quantification of occludin protein expression in CTX and SC of young (red columns) and old (blue columns) mice. Columns represent different MW bands of the protein. Western blots from which expression was quantified are shown for **e** CTX and **f** SC. **g** Quantification of tubulin beta-3 protein expression in CTX and SC of young (red columns) and old (blue columns) mice. Western blots from which expression was quantified are shown for **h** CTX and **i** SC. Analyses of claudin-5, occludin, and tubulin beta-3, protein expression in **j** brain ECs and **k** CTX and **l** HIP from mice of different ages. Proteomics data was obtained from Todorov-Volgyi et al., 2024 **j** and Tsumagari et al., 2023 **k, l**

We quantified Occludin and Claudin-5 protein levels in CTX and SC by Western blot (Fig. 5a-f). All blots were stained with Revert total protein stain and there were no significant differences between ages, indicating equivalent amounts of protein were loaded and transferred (Supplementary Fig. S1-S2).

Claudin-5 is the predominant claudin protein in mouse and human brain ECs. It has a MW of 23 kDa (uniprot.org/uniprotkb/O54942/entry) and is post-translationally modified [37]. To include potential PTMs we quantified bands in the ~ 25–37 kDa range. Degradation of claudin-5 is regulated by the ubiquitin–proteasome system (UPS) and is polyubiquitinated resulting in molecular weights of up to ~ 100 kDa [38]. To capture potentially ubiquitinated Claudin-5, the high MW ~ 75–100 kDa bands were also quantified. In CTX, there was a significant decrease in abundance of the ~ 75–100 kDa bands (*p* = 0.016, Fig. 5a, b), but only a trend toward an increase for the ~ 25–37 kDa bands (*p* = 0.19). In SC, there was a significant ~ 2.5-fold increase in old compared to young for the low MW ~ 25–37 kDa bands (*p* = 0.016, Fig. 5a, c). There was a significant ~ 2.8-fold decrease in high MW Claudin-5 in old compared to young in SC (*p* = 0.016).

Occludin is an integral protein in brain EC tight junctions, although its function remains inconclusively resolved. Occludin has a molecular weight (MW) of ~ 59 kDa (uniprot.org/uniprotkb/Q61146/entry), but has multiple phosphorylation sites and as the antibody detects lower MW bands in addition to the main band, we quantified all bands. In CTX, there was no significant effect off aging on abundance of the main band at ~ 60 kDa, (*p* = 0.556, Fig. 5d, e). There was a trend toward a decrease (*p* = 0.063) in the old group for the higher MW, ~ 75 kDa, and no significant age effects for the ~ 100 kDa band (*p* = 0.19). Similarly, the lower MW ~ 45 kDa and ~ 37 kDa bands were not affected by age (*p* = 0.73 and *p* = 0.905, respectively). When all MW bands were considered together, there was not a significant difference between age groups (*p* = 0.111). In SC, with the exception of a significantly decreased abundance of the high MW ~ 100 kDa band in the old group (*p* = 0.016), there were no other significant changes (Fig. 5d, f). Tubulin beta-3 was also used as a reference protein and there were no significant age-related differences in Tubulin beta-3 expression (Fig. 5g-i).

To more comprehensively assess effects of aging on EC tight junction-related protein expression, enrichment analyses were carried out using the 70-gene EC junction gene set and the proteomics data from the Tsumagari et al. study [23]. Tsumagari et al., identified 7,168 proteins in CTX and HIP of 3, 15, and 24 month-old, C57BL6J male mice (the same sex and strain as used in the current study). Of the 70 EC junction genes, only two overlapped (Frmd4a, Tjp2) with the gene names for the 187 differentially expressed (FDR q < 0.05) proteins in CTX, there was no significant enrichment (hypergeometric *p* = 0.101). In HIP the overlap was also two genes (Dig1, Tjp2) with the 244 differentially expressed proteins. Again, there was not significant enrichment (hypergeometric *p* = 0.156). Of note, while Ocln protein was detected in both CNS regions and was unchanged, Cln5 was not detected. Expression of Tjp2 protein was significantly increased in both CNS regions with age.

As our genomics analyses indicated significant barrier dysfunction (see Fig. 1.d), we compared the 136-gene barrier dysfunction gene set with the CTX and HIP differentially expressed proteins. In CTX, there were 9 overlapping genes (Anln, Anx1, Dpysl3, Lamb1, Lgals, Marcksl1, S100a6, Tnc, Vcan), resulting in a significant enrichment (hypergeometric *p* = 1.11e-06). In HIP, there were 7 overlapping genes (Anln, Dpysl3, Flnc, Pdlim1, S100a6, Tnc, Tubb6), also resulting in a significant enrichment (hypergeometric *p* = 0.00045).

The proportion of ECs in the CNS is relatively low compared to other cell types. Therefore, as for genomics, proteomics investigations based on tissue homogenates can suffer detection limitations for low abundance proteins expressed in minority cell types. This may be why the Tsumagari study did not reliably detect Cldn5. To address this limitation, we made use of a proteomics resource generated from purified brain ECs [24] (available at http://becaging.de/). This study analysed brain EC protein expression at 3, 6, 12, and 18 months of age and identified 4,137 proteins. With adult, 3 month-old mice as the reference, the abundances of 850 proteins were altered by aging (ANOVA, *p* < 0.05) with 356 proteins surviving correction for multiple comparisons (FDR q < 0.05) [24]. There were no significant effects of aging on Ocln (12 v 3 mos, *p* = 0.599; 18 v 3 mos, *p* = 0.78), or on Cldn5 protein expression (12 v 3 mos, *p* = 0.904; 18 v 3 mos, *p* = 0.853).

Using gene names for the 356 differentially expressed proteins (FDR < 0.05 for any age comparison to the 3-month age group), there were only 2 overlapping genes (Ctnnb1, Vasp) with the EC junction gene set, and no significant enrichment (hypergeometric *p* = 0.273). Even when dropping FDR for the 18 v 3-month comparison and using the less stringent *p* < 0.05, only 4 genes overlapped (Ctnnb1, Pdcd6ip, Strn, Vasp), enrichment again did not reach significance (hypergeometric *p* = 0.161). To improve likelihood of identifying even subtle effects at any age, all age comparisons were assessed using a *p* < 0.05 (no FDR). Nine genes overlapped (Ctnnb1, Mpdz, Mpp5, Pdcd6ip, Strn, Tbcd, Tjp1, Tjp2, Vasp), with enrichment reaching significance (fold-enrichment 3.46; hypergeometric *p* = 0.001). Decreased expression of junction proteins Ctnnb1, Tjp1,Tjp2, and Vasp was found for the 6 versus 3 month comparison, whereas decreases at old ages only occurred for Ctnnb1 and Vasp. Significant increases in protein expression were found at 6 months for Mpdz and Mpp5, and at old ages for Pdcd6ip, Strn, and Tbcd. Assessing for enrichment of the barrier dysfunction gene set showed little overlap, with only 4 of the 136 dysfunction genes overlapping with the 735 differentially expressed EC proteins (18 v 3 mos comparison, *p* < 0.05), resulting in no significant enrichment (hypergeometric *p* = 0.608). Overall, the protein analyses generally support the genomics outcomes.

### Aging subtly affects CNS blood vessel density but not pericyte coverage

**Fig. 6:**
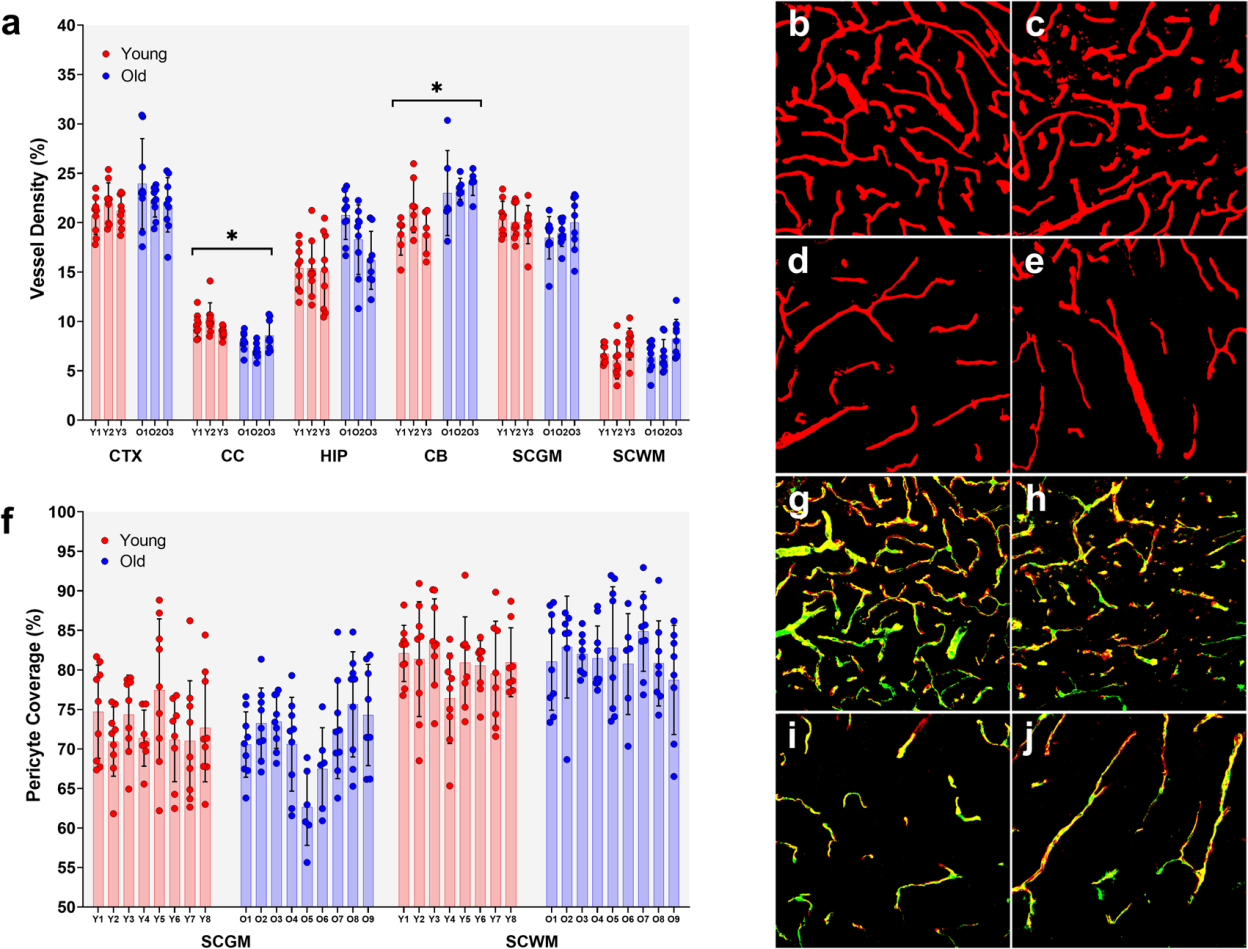
Blood vessel density and pericyte coverage in aging mouse CNS. **a-e** Blood vessel density. **a** Vessel density was quantified by Col-IV immunolabelling across 6 CNS regions. Points are individual measurements (6–9/animal). Bars are mean and error bars are ± SD. *n* = 3/gp/region. * Nested t-test *p* < 0.05. **b-e** False colour processed images of Col-IV immunolabelling (red) in young **b, d** and old **c, e** spinal cord grey **b, c** and white **d, e** matter at 40x. **f-j** Pericyte coverage. **f** Pericyte coverage was quantified using Col-IV and CD13 double immunolabelling. Points are individual measurements (6–9/animal). Bars are mean and error bars ± SD. *n* = 8–9/gp/region. **g-j** False colour processed images of Col-IV (red) and CD13 (green) double (yellow) immunolabelling in young **g, i** and old **h, j** spinal cord grey **g, h** and white **i, j** matter at 40x. CTX – cortex; CC – corpus callosum; HIP – hippocampus; CB – cerebellum; SC – spinal cord; GM – grey matter; WM – white matter

Blood vessel density was assessed using Col-IV immunolabelling to identify vessels. The expected lower blood vessel density in WM compared to GM was observed [39], with density in WM being ~ 50% of that observed in GM regions (Fig. 6a-e). Effects of aging were subtle, and only seen in two regions, with a small decrease in density in the CC (*p* = 0.041) and a small increase in the CB (*p* = 0.027). CTX, HIP, SCGM, and SCWM all showed no significant effects of aging on blood vessel density.

Pericyte coverage of blood vessels was assessed in the SC. Immunolabelling for CD13 was used to identify PCs and Col-IV immunolabelling to identify blood vessels (Fig. 6g-j). There were no significant differences between age groups in pericyte coverage in either the SCWM or SCGM (Fig. 6f). Pericyte coverage of blood vessels in WM appeared higher than in the GM, as expected [40].

### Functional analyses of blood-CNS barrier permeability reveal general lack of age effects

#### Age-related loss of CNS water content

**Fig. 7:**
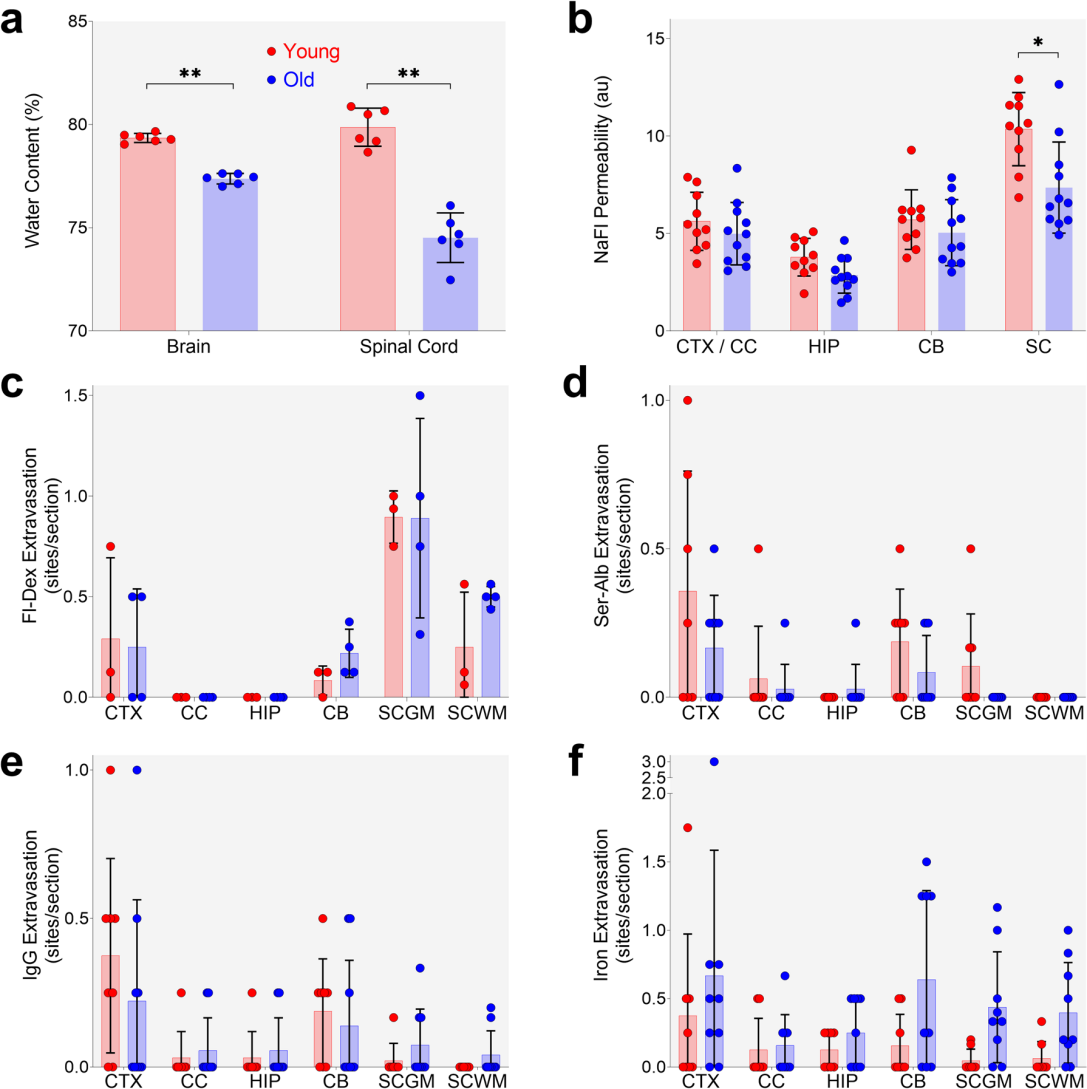
Functional assessment of the impact of aging on blood-CNS barrier permeability. **a** Effects of aging on brain and spinal cord water content expressed as percent of wet weight. *n* = 6/gp. **b** Sodium Fluorescein (NaFl) permeability. *n* = 10–11/gp. **c** Fluorescein dextran (Fl-Dex) permeability. *n* = 3–4/gp. **d** Serum albumin (Ser-Alb) permeability. *n* = 8–9/gp. **e** Immunoglobulin G (IgG) permeability. *n* = 8–9/gp. **f** Iron labelling for microhaemorrhages. *n* = 8–9/gp. Graphs: points represent individual animals; bars are mean and error bars are ± SD; * one-tailed Wilcoxon Mann–Whitney U Exact test corrected *p*-value < 0.05. Legend in **a** holds for all graphs. CTX – cortex; CC – corpus callosum; HIP – hippocampus; CB – cerebellum; SC – spinal cord; GM – grey matter; WM – white matter

Brain and SC water content were determined using oven drying. Wet weight and dry weight both significantly increased with age in both regions (Supplementary Fig. S3a-b). Water content, expressed as a percentage of total (wet) weight, significantly decreased in the aged brain and SC (Fig. 7a).

#### No age-related increase in blood-CNS barrier tracer permeability

BBB and BSCB permeability were measured using the low molecular weight tracer sodium fluorescein (NaFl, MW ~ 376 Da). CNS tissue and serum levels of NaFl were determined by fluorometry. Tissue levels were normalised to serum NaFl for each sample. Permeability did not change significantly in CTX, CC, or the CB, whereas it decreased in the SC (*p* = 0.01) (Fig. 7b). The apparent decrease in the HIP did not survive correction for multiple comparisons (*p* = 0.018 uncorrected, *p* = 0.054 corrected).

Permeability was also measured using fluorescein dextran (MW ~ 3 kDa), a molecular weight tracer in the size range of small proteins. The number of areas demonstrating leakage (extravascular dextran) per tissue section was counted for CTX, CC, HIP, cerebellum grey matter (CBGM), cerebellum white matter (CBWM), SCGM and SCWM. Leakage indications were rare (Fig. 7c, and Supplementary Fig. S4a-d for images) and there were no significant age-related differences in any CNS region investigated.

#### No age-related increase in endogenous indicators of CNS barrier dysfunction

We also investigated barrier permeability by labelling for endogenous indicators of barrier dysfunction. Brain and SC sections were immunolabelled for serum albumin (MW ~ 68kDA) and IgG (MW ~ 150kDA), and the number of areas with perivascular serum albumin (Fig. 7d) or IgG (Fig. 7e) per tissue section were counted. In both cases, perivascular labelling was rare (as for dextran experiments), and no regions had significant age-related effects (see Supplementary Figs. S5a-d and S6a-d for albumin and IgG images, respectively).

We also assessed barrier permeability by staining brain and SC sections using Prussian Blue to label iron, a marker of microhaemorrhages. Putative microhaemorrhage events were rare, as for serum albumin and IgG perivascular labelling, and similarly there were no age-related effects in the regions investigated (Fig. 7f, and Supplementary Fig. S7a-d for images). We did find an age-related effect in the thalamus (Supplementary Fig. S8a-b) with iron detected in old animals, which has previously been reported [41, 42] and serves as a positive control for the labelling.

## Discussion

Blood-CNS-barrier disruption is thought to contribute to age-related decline in CNS function. However, a consensus on the effects of normal, non-pathological, aging on barrier integrity is yet to be reached and the issue remains highly controversial [6]. To help resolve this complex issue, we have carried out a comprehensive assessment of barriers across CNS regions, using an array of molecular, including a meta-analysis of scRNA-seq studies, protein, and functional analyses.

Transcriptomic profiling and enrichment analyses indicated the three cell types most directly influencing barrier function, ECs, PCs, and astrocytes, were affected by aging. To more directly assess barrier properties at the molecular level, we did an enrichment analysis with an EC junction gene set [30], but found no significant enrichment, indicating aging does not to a broad degree affect barrier EC junctions in CTX or SC. However, when enrichment was analysed with an EC dysfunction gene set, derived from conditions associated with barrier leakiness [30], there was significant enrichment for both CTX and SC. Note, only one gene is common to both EC junction and dysfunction gene sets [30]. Overall, this suggests aging makes CNS ECs dysfunctional but not necessarily leaky. Indeed, our scRNA-seq meta-analysis is consistent with this notion as we found little evidence for EC junction changes, yet a number of GOs were significantly impacted by aging, in particular those involving the immune system.

qPCR analyses also revealed variable effects of aging across the CNS, with the SC being the most affected overall, and HIP the least affected. The limited impact on the HIP is somewhat surprising given its known susceptibility to aging. Studies have reported aging-related hippocampal atrophy, functional decline [43, 44], and impaired hippocampal-dependent cognitive performance [45]. Our findings suggest the hippocampal BBB is not a contributing factor to aging-related cognitive decline, which is consistent with recent human work that showed no correlation between age and indices for PC dysfunction and hippocampal BBB leakiness in either normal or mildly cognitively impaired cohorts [46].

EC junction gene qPCR analysis found just one significant change with age, with Tjp1/ZO-1 expression increased only in CB. Other studies have reported age-related decreases [19, 21, 47, 48], or no change [18, 48] in expression of EC junction genes/proteins. Our RNA-seq enrichment analyses and our scRNA-seq meta-analysis, support our qPCR finding of minimal tight junction changes. A recent brain EC proteomics study also did not find widespread age-related changes in junction-related protein abundances [24]. Overall, decreased CNS EC junction gene/protein expression appears not to be a widespread characteristic of normal aging.

For PCs, expression of the barrier-critical gene, Pdgfrb, was only significantly decreased in CTX. Expression of the heparin-binding, ECM, leucine-rich repeat protein (Prelp) was increased in CC and SC GM (and CTX *p* = 0.06, SC WM *p* = 0.05) of old mice. Blood-CNS-barrier function for Prelp is not known, but it does bind soluble complement inhibitor C4b-binding protein [49], and may therefore be involved in the innate immunity complement response aspect of inflammaging. Interestingly, we found decreased pleiotrophin (Ptn) expression in SC GM and WM. Ptn is a PC-secreted neurotrophic factor that when administered exogenously to mice prevents the neuronal loss associated with PC degeneration [50]. While Ptn loss alone is not thought to lead to vascular disturbances or neuronal loss in young healthy animals [50, 51], it may be more deleterious in the context of the inflamed, aging CNS.

Eight EC – PC signalling genes were also investigated, and while transforming growth factor beta (Tgfb) signalling was significantly age-affected, our laser microdissection data (Supplemental Table S9) and single-cell data (Online resource 2) suggest the Tgfb signalling is not microvessel derived.

Genes involved in immune cell invasion were generally increased in expression in old animals, with Icam-1 being the most markedly affected (up ~ fivefold) and most widely affected (5 regions). Icam-1 is expressed by ECs and is involved in leukocyte adhesion and transmigration into CNS parenchyma [52]. Notably, Icam-1 expression is increased in the inflamed CNS, suggesting peripheral immune cells may infiltrate. Based on molecular signatures, we (and others) have found the mouse CNS to be inflamed, however others have not observed markers suggesting an increased number of immune cells have infiltrated [19, 53]. Icam-1 is not the only molecule involved in leukocyte transmigration into the CNS and the increased expression we report here may be indicative of a priming event that requires additional inflammatory factors before immune cells can extravasate. Others have reported increased numbers of infiltrating immune cells in the aged CNS, but also reported increased permeability to an injected dextran and decreased gene expression of Cdh5, Ocln, Tjp1, and Cldn5 [54]. We did not see increased permeability to a smaller dextran, nor did we find robust decreased expression of EC junction genes.

We then determined the time course of changes across the adult mouse lifespan. We quantified expression of a subset of genes that represented multiple aspects of barrier cell types and function in the SC, the most affected region as determined in qPCR analyses, and an understudied CNS region in aging research. Of the 12 genes assessed, only three were significantly changed and were overexpressed at the oldest age only, with all three being immune cell invasion related (Cxcr4, Emp1, Icam-1). Overall, these age-course data indicate that age-related changes in the BSCB occur in the latter half of the adult life span.

As bulk sequencing of RNA extracted from tissue homogenates suffers from loss of cell-type specificity, we carried out a meta-analysis of single cell sequencing studies of the aged mouse brain to assess the impacts of aging on gene expression in ECs and PCs (Fig. 4). As we saw with the enrichment analyses of bulk sequencing data, aging impacted gene expression in both ECs and PCs, although the latter were more impacted, based on number of DEGs. There was little evidence for impact of aging on EC junction genes, although expression of Septin2, which is thought to be important for EC junction organisation and barrier integrity [33], increased with age. The age-related drop in EC connexin-43 (Cx43) expression is interesting given the recent report showing its role in maintaining BBB integrity, not via its gap-junction properties, but via a poly(ADP-ribose) polymerase 1 mediated mechanism [32]. EC Cx43 protein expression in the Zhan study was reduced by > 50%, which may explain the increased BBB permeability. Variability in CNS EC Cx43 expression may be one factor contributing to differences in barrier permeability between studies. Expression of genes involved in signalling between ECs and PCs was not altered by aging but in the meta-analysis, but basement membrane genes were. When viewed from a GO perspective, ECs and PCs differed markedly, with the former enriched for immune functions and the latter for blood vessel homeostasis. Overall, the meta-analysis largely supported the bulk sequencing analyses, but with the advantage of cell-typespecific context.

In summary, our molecular data yielded limited support for perturbation of tight junctions between ECs, although aging did impact each of the cell types that comprise the blood-CNS barriers, possibly establishing a ‘primed’ state. Functional implications, as assessed by biological processes gene ontology enrichment, indicated expression of genes involved in signalling between barrier cells and immune cell invasion were the most widely and markedly affected, whilst the molecular effects appear to be occurring relatively late in adult life. Overall, the effects of aging on gene expression were typically modest, so we then determined impacts at the protein level.

Traditional Western blotting yielded an interesting pattern of high and low molecular weight bands for claudin-5, in particular in SC. Non-modified claudin-5 has a MW of ~ 23 kDa, and we detected low MW bands at 25–37 kDa, presumably indicating phosphorylated protein [37]. There were also abundant high MW bands for claudin-5 at 75–100 kDa, which is likely polyubiquitinated claudin-5 [38]. Old age was associated with a much greater abundance of the low MW protein compared to the young age group and concomitantly, lower abundance of the high MW protein. One possible explanation for this signature is reduced claudin-5 turnover, due to age-related decline in the ubiquitin–proteasome and autophagy-lysosomal systems, the major protein degradation pathways that are known to be responsible for the decline in cell proteostasis, a major hallmark of aging [55]. For occludin (MW 59 kDa), there were no significant age effects on abundance of the ~ 60 kD band in either CNS region, but there were declines for high MW bands. Given the poorly understood roles of CNS occludin, it is not obvious what the effects of decreased high MW occludin might be.

To capture a much larger proportion of the proteome, we made use of two recently published proteomics studies that investigated the effects of aging on the mouse CTX and HIP proteomes [23], and specifically on the mouse brain EC proteome [24]. We did not detect enrichment for an EC junction gene set in the whole brain proteome data but did for a barrier dysfunction gene set, again supporting our genomics data indicating minimal age-related perturbation of EC junctions but general barrier dysfunction similar to that observed in neurodegenerative disorders [30]. In the brain EC proteome, there were no significant effects on occludin or claudin-5, although post-translational modifications of these proteins were not reported. There was modest enrichment for the EC junction gene set, but only when the stringent criterion of multiple comparison correction was removed from the proteomics data. Even then, decreases in tight junction protein abundance were only seen at 6 months and not older ages.

The genomics and proteomics data essentially indicate the same outcome, the impacts of aging on the blood-CNS-carriers are modest, and on EC junctions, are minimal. We then determined if morphological and/or functional characteristics of the blood-CNS barriers were impacted.

Previous studies have suggested blood vessel density is reduced in the aged CNS [14, 48, 56], although others have reported no change [3, 18, 21]. While we found the expected WM vessel density being approximately 50% of that in GM, there was little change in density with aging, the exception being a decrease in CC, and increase in CB (Fig. 6a-e). Thus, we do not observe the changes previously seen, where marked decreases in vessel density were reported [14, 56]. In a recent analysis of the cerebrovasculature of whole mouse brain using modern imaging techniques, it was found that early (18 months) age-related changes in vessel density were relatively modest (< 10%), region- and cortical layer-dependent, and generally only reached significance due to the age-related increase in brain volume [57]. This study highlights the modest and highly variable nature of the aging-related changes in the cerebrovasculature and may shed light as to why there are so many conflicting reports regarding vessel density.

Another factor in barrier function is PC coverage, and our molecular data indicates potential for PC dysfunction with aging. Consistent with previous reports, we did not find age-related loss of PC coverage [2, 3, 14, 18], although we restricted our analysis to SC. We did find PC coverage appeared greater in WM, consistent with previous reports [40]. There is inconsistency across studies with reports of decreased pericyte coverage with aging [20, 21]. Again, the Bennett study, having the advantage of a whole-brain analysis, may help explain this inconsistency. As for vessel density, they found pericyte density in old brains was within 10% of that in young brains and was not significantly different. The exceptions to this were basal forebrain and deep cortical layers showing decreased density, and superficial cortical layers showing increased pericyte density with age [57]. With such brain regional and cortical layer-dependent effects, in combination with the modest effect sizes, it is little wonder there have been conflicting studies regarding pericyte density.

Taken together, our vessel density and PC coverage data are largely consistent with those of arguably the most comprehensive analysis of the aging mouse cerebrovasculature to date [57]. Both studies indicate aging does not have marked, CNS-wide impact on microvessel gross structure, suggesting blood vessels are mostly intact. There are, of course, exceptions to this, with phenomena such as age-related increased vessel tortuosity and decreased branching (we did not measure either of those). While gross cerebrovascular changes may not be widely evident, there are well-established ultrastructural changes associated with aging. These include increases in basement membrane thickness, PC to EC contact, EC pseudopod number, tight junction tortuosity, and changes in PC and EC mitochondrial number and volume [58, 59]. These ultrastructural barrier changes may have functional sequelae. While our molecular, proteomic, and morphometric analyses indicating relatively limited impact of aging on blood-CNS-barriers, we carried out a number of complementary functional analyses. Tissue water content is one measure of barrier permeability. Increased tissue water is indicative of barrier leakiness [60]. Contrary to expectation, we found significant decreases in water content in both the brain (down ~ 2%) and spinal cord (down ~ 5%), which may warrant further investigation of aging CNS water transport mechanisms, particularly Aqp4 function, which we found to be moderately reduced in our RNA-sequencing data, but not in our single cell analysis nor in an aging CTX and HIP mass spectrophotometry dataset [23]. This decreased water content is in stark contrast to data from Park et al. who reported a ~ 4% increase in brain water content with age [14]. We found a number of differences between ours and the Park study. For example, they found an ~ 50% decrease in cortical microvessel density, whereas we found no age-related changes. They found a significant decrease in brain maximum width while we did not (Supplemental Fig. S9). They also found increased extravascular NaFl whereas we did not. Our data are consistent with a previous report of decreased extracellular space with age [61].

Exogenous tracers of different molecular weights were also used to assess barrier permeability. We found no effects of age with relatively small (NaFl) and large (dextran) molecular weight tracers, with the exception of a significant decrease in extravasated NaFl in SC of old animals. This decrease is likely not due to a decrease in vessel density as we did not find an age effect on density in SC. Consistent with previous reports, we did find the BSCB to be more permeable than the BBB [12, 40]. Ritzel et al. [12] also found a similar SC outcome, with lower NaFl permeability in aged mice [12]. Speculatively, this decreased permeability may be associated with the increased low MW claudin-5 protein abundance we found in the old age group. Lack of claudin-5 leads to a size-selective permeability (< 800 Da) of the BBB [62], so increased claudin-5 could effectively block more NaFl from entering the CNS parenchyma. Park et al. (2018) [14] found an ~ 50% increase in brain NaFl permeability in old mice, yet Goodall et al. [3] found no age-related increase in Evans blue permeability. The reasons for the differences between studies investigating extravasation of exogenous tracers are difficult to pin point. Variables include the nature of the tracer and how it is quantified and normalised (e.g. to levels in the blood), route and duration of tracer administration, use of animal anaesthesia (e.g. isoflurane can increase tracer extravasation [63–65]). Until these variables are understood it is unlikely there will be a consensus on how aging impacts the blood-CNS-barriers.

Exogenously administered barrier tracers may not recapitulate the physiology of endogenous blood-borne factors [21]. Therefore, we also analysed the degree to which endogenous IgG, plasma albumin, and hemoglobin iron (an indicator of microhemorrhage) localised extravascularly across CNS regions. As with the exogenous tracers, there were no increases in extravascular labelling with aging. One exception was increased iron labelling in the thalamus of old mice, which is consistent with previous reports of increased microbleeds in the aged thalamus [41, 42]. A number of studies have reported increased extravascular labelling of endogenous proteins in aged mice [10, 16, 19, 57, 66]. Yet, other work demonstrated age-related increases in endogenous protein leakage was dependent on decreased pericyte coverage [18]. As we did not find decreased pericyte coverage in aged CNS, nor evidence of extravascular proteins, our data are consistent with those of Bell et al., and for the requirement of pericyte decline for this phenomenon to occur. Measuring soluble Pdgfrb [46] or other indicators of pericyte damage may be an ideal biomarker that could be reported in blood-CNS-barrier studies to help understand inconsistencies between studies.

In summary, our functional findings support our molecular, protein, and morphometric data, and indicate blood-CNS barriers do not get leakier with aging, at least not via the paracellular pathway. If anything, the BSCB becomes less permeable to small molecules, as indicated by our tracer, claudin-5, and water content data. Aging-related increases in basement membrane thickness [58] could contribute to loss of permeability. Basement membrane is made up of extracellular matrix proteins secreted by the three component cell types of the barriers. Aging-related changes in basement membrane thickness and/or relative abundances of extracellular matrix proteins could affect barrier permeability.

## Conclusions

In conclusion, our data does not support a loss of barrier tight junctions in aging CNS, but they do indicate development of an inflammatory profile that may render the barriers susceptible to CNS and/or peripheral inflammatory events. In other words, normal aging may constitute a molecular ‘first hit’, with additional insults required for a barrier breach, at least by the paracellular pathway. This seems to be the case for cerebral microhemorrhages 74, 75. Whatever the reasons for the discrepancies between ours and previous findings, there remains much to do before the effects of aging on the blood-CNS-barriers are fully understood. Indeed, recent work using a novel ‘endogenous tracer’ approach where blood proteins were labelled, demonstrated the transcellular pathway is impacted by aging [21], indicating the two pathways from the circulation into the CNS parenchyma maybe differentially affected by aging.

The interstudy inconsistencies, which have been recently discussed in an excellent review [6], have generated much controversy in the field. Arguably, the field has been polarised into the ‘leakers’ and ‘non-leakers’, views that could be obscuring an opportunity to better understand factors involved in age-related barrier breakdown. There are many potentially confounding variables to consider when trying to determine why the barriers fail with aging. This is in addition to the obvious ones of species, strain, sex, CNS region, and age (see Table 1 in [6] for overview).

Other, not necessarily mutually exclusive, factors that could be considered and reported in blood-CNS-barrier studies include:

1. Diet type – diet is known to affect barrier function, with diets enriched in saturated fatty acids or cholesterol showing increased serum IgG extravasation [67]. The gut micobiota can also affect blood-CNS-barrier permeability either directly with bacterial metabolites, or indirectly through peripheral immune cell activation [68, 69]. For example, it has been shown that short chain fatty acids produced by the gut microbiota affect the function of astrocytes and microglia [70], which could in turn alter CNS barrier permeability.
2. Inflammation – aging is characterised by low grade chronic, sterile inflammation (inflamaging) and inflammation affects barrier permeability [54]. A battery of inflammatory biomarkers for the blood and CNS tissue would be informative in gauging the degree of inflammation in individual animals and its association with barrier leakiness in each animal. For example measuring blood and CNS complement component, C3, levels could indicate cerebrovascular inflammation [54].
3. Barrier markers – as alluded to above, indicators such as soluble Pdgfrb (sPdgfrb) could be useful in determining barrier dysfunction in individual animals [46, 71]. Although, having to measure this biomarker in CSF could be a limitation to widespread use. Also age-related increases in brain EC Vcam1 expression is associated with neuroinflammation. Elevated EC Vcam1 expression leads to increased shedding of soluble Vcam1 (sVcam1) into plasma and, therefore, is an indcator of EC inflammation and potentially barrier dysfunction [13]. sVcam1 and sPdgfrb could be valuable plasma biomarkers for blood-CNS-barrier dysfunction.
4. Metabolite levels – nicotinamide adenine dinucleotide (NAD +) is a key player in cellular metabolism [72] and low NAD + levels were recently shown to be associated with barrier leakiness [32]. By reporting levels of NAD + and/or related metabolites, it will be possible to correlate measures with other barrier-related factors.

Other considerations include standardisation of techniques for measuring barrier permeability, such as exogenous tracer types, routes and duration of administration, normalisation methods, use and type of anaesthesia.

As mentioned, there is much to understand regarding blood-CNS-barrier function in the aging CNS. The discrepancies between studies should not be considered controversial, but as an indication of our inadequate understanding and as a basis to explore broader possibilities.

## Limitations

There are important limitations to our experiments that need to be considered when interpreting our datasets. Below we outline the major potential limitations of our study:

We have only included male mice, not an uncommon limitation in the field. Given the reports of sex differences in blood–brain barrier function (e.g. [73]), inclusion of female mice to these types of study is necessary to achieve a comprehensive understanding of the effects of sex. Some consider use of mice as models of human aging to be problematic. For practical reasons and to allow mechanistic approaches, the use of preclinical models is necessary.

The use of bulk tissue for most molecular and some protein analyses is a key limitation. Although the use of bulk tissue for RNA-sequencing and qPCR, and Western blotting provides robust measurements, spatial information is lost and local effects that may occur in a minority of cells can be averaged out. This is an important consideration for examining the BBB and BSCB, as age-related effects in gene/protein expression or vascular leakage may only occur at particular foci in the vasculature. We have attempted to address these technical limitations in a number of ways. For molecular analyses, we complemented our experiments in bulk tissues with laser microdissection to limit expression measurements to barrier cells, and with a meta-analysis of aging brain single cell studies where expression can be attributed to an individual cell, although expression measurements are less robust. We also used brain proteome and brain EC proteome resources to complement our protein work. Importantly, results from all experiments supported the same conclusion – that EC junction components are predominantly not impacted by aging in the CNS.

Limitations of our anatomical approaches, such as measurement of blood vessel density and pericyte coverage, are the sampling and limited number of CNS regions. A recent extensive analysis of the effects of aging on the vasculature across the whole mouse brain provides an important example of not only regional but also cortical layer-dependent effects [57], and demonstrates how limited sampling can impact outcomes.

A limitation of our functional analyses is the use of ex vivo rather than in vivo measurements. Although our ex vivo measurements have disadvantages, as with all ex-vivo analyses, they also offer a major advantage over in vivo approaches, since they do not require potentially inflammation-inducing surgery or anaesthesia.

## Supplementary Information

Below is the link to the electronic supplementary material.

Supplementary file1 Online resource 1 Detailed methods (DOCX 42 KB)

Supplementary file2 Online resource 2 Bulk RNA-seq results. (XLSX 4746 KB)

Supplementary file3 Online resource 3 Single cell DEGs for all clusters. (XLSX 610 KB)

Supplementary file4 (XLSX 37 KB)

Supplementary file5 (XLSX 93 KB)

Supplementary file6 (DOCX 19397 KB)

## Data Availability

All data is available upon request to the authors. RNA sequencing reads are available in BioProject accession number PRJNA1073059. Supplemental and supporting data can be accessed at https://figshare.com/collections/Aging_disrupts_blood-brain_and_blood-spinal_cord_barrier_homeostasis_but_does_not_increase_paracellular_permeability/7041869

## Abbreviations

BBB: Blood-brain barrier
BSCB: Blood-spinal cord barrier
CB: Cerebellum
CC: Corpus callosum
CNS: Central nervous system
ColIV: Collagen IV
CSF: Cerebrospinal fluid
CTX: Cortex
DEG: Differentially expressed gene
EC: Endothelial cell
Fl-Dex: Fluorescein dextran
GM: Grey matter
GO: Gene ontology
HIP: Hippocampus
IgG: Immunoglobulin G
NaFl: Sodium fluorescein
PBS: Phosphate buffered saline
PC: Pericyte
PFA: Paraformaldehyde
SC: Spinal cord
scRNA-seq: Single cell RNA-sequencing
Ser-Alb: Serum albumin
SMC: Smooth muscle cell
WM: White matter

## References

[1] Montagne A, Barnes SR, Sweeney MD, Halliday MR, Sagare AP, Zhao Z, et al. Blood-brain barrier breakdown in the aging human hippocampus. Neuron. 2015;85:296–302. 25611508 10.1016/j.neuron.2014.12.032 PMC4350773

[2] Bors L, Toth K, Toth EZ, Bajza A, Csorba A, Szigeti K, et al. Age-dependent changes at the blood-brain barrier. A Comparative structural and functional study in young adult and middle aged rats. Brain Res Bull. 2018;139:269–77. 29522862 10.1016/j.brainresbull.2018.03.001

[3] Goodall EF, Wang C, Simpson JE, Baker DJ, Drew DR, Heath PR, et al. Age-associated changes in the blood-brain barrier: comparative studies in human and mouse. Neuropathol Appl Neurobiol. 2018;44:328–40. 28453876 10.1111/nan.12408 PMC5900918

[4] Abbott NJ, Patabendige AA, Dolman DE, Yusof SR, Begley DJ. Structure and function of the blood-brain barrier. Neurobiol Dis. 2010;37:13–25. 19664713 10.1016/j.nbd.2009.07.030

[5] Daneman R, Prat A. The blood-brain barrier. Cold Spring Harb Perspect Biol. 2015;7:a020412. 25561720 10.1101/cshperspect.a020412 PMC4292164

[6] Banks WA, Reed MJ, Logsdon AF, Rhea EM, Erickson MA. Healthy aging and the blood-brain barrier. Nat Aging. 2021;1:243–54. 34368785 10.1038/s43587-021-00043-5 PMC8340949

[7] Garton MJ, Keir G, Lakshmi MV, Thompson EJ. Age-related changes in cerebrospinal fluid protein concentrations. J Neurol Sci. 1991;104:74–80. 1717663 10.1016/0022-510x(91)90218-v

[8] Pakulski C, Drobnik L, Millo B. Age and sex as factors modifying the function of the blood-cerebrospinal fluid barrier. Med Sci Monit. 2000;6:314–8. 11208329

[9] Chen RL. Is it appropriate to use albumin CSF/plasma ratio to assess blood brain barrier permeability? Neurobiol Aging. 2011;32:1338–9. 19709781 10.1016/j.neurobiolaging.2008.08.024

[10] Senatorov Jr VV, Friedman AR, Milikovsky DZ, Ofer J, Saar-Ashkenazy R, Charbash A, et al. Blood-brain barrier dysfunction in aging induces hyperactivation of TGFbeta signaling and chronic yet reversible neural dysfunction. Sci Transl Med. 2019;11. 10.1126/scitranslmed.aaw8283 31801886

[11] Montagne A, Nation DA, Sagare AP, Barisano G, Sweeney MD, Chakhoyan A, et al. APOE4 leads to blood-brain barrier dysfunction predicting cognitive decline. Nature. 2020;581:71–6. 32376954 10.1038/s41586-020-2247-3 PMC7250000

[12] Ritzel RM, Patel AR, Pan S, Crapser J, Hammond M, Jellison E, et al. Age- and location-related changes in microglial function. Neurobiol Aging. 2015;36:2153–63. 25816747 10.1016/j.neurobiolaging.2015.02.016

[13] Yousef H, Czupalla CJ, Lee D, Chen MB, Burke AN, Zera KA, et al. Aged blood impairs hippocampal neural precursor activity and activates microglia via brain endothelial cell VCAM1. Nat Med. 2019;25:988–1000. 31086348 10.1038/s41591-019-0440-4 PMC6642642

[14] Park MH, Lee JY, Park KH, Jung IK, Kim KT, Lee YS, et al. Vascular and neurogenic rejuvenation in aging mice by modulation of ASM. Neuron. 2018;100:762. 30408445 10.1016/j.neuron.2018.10.038

[15] Hafezi-Moghadam A, Thomas KL, Wagner DD. ApoE deficiency leads to a progressive age-dependent blood-brain barrier leakage. Am J Physiol Cell Physiol. 2007;292:C1256-1262. 16870825 10.1152/ajpcell.00563.2005

[16] Nyul-Toth A, Tarantini S, DelFavero J, Yan F, Balasubramanian P, Yabluchanskiy A, et al. Demonstration of age-related blood-brain barrier disruption and cerebromicrovascular rarefaction in mice by longitudinal intravital two-photon microscopy and optical coherence tomography. Am J Physiol Heart Circ Physiol. 2021;320:H1370–92. 33543687 10.1152/ajpheart.00709.2020 PMC8260380

[17] Stamatovic SM, Martinez-Revollar G, Hu A, Choi J, Keep RF, Andjelkovic AV. Decline in Sirtuin-1 expression and activity plays a critical role in blood-brain barrier permeability in aging. Neurobiol Dis. 2019;126:105–16. 30196051 10.1016/j.nbd.2018.09.006 PMC6401345

[18] Bell RD, Winkler EA, Sagare AP, Singh I, LaRue B, Deane R, et al. Pericytes control key neurovascular functions and neuronal phenotype in the adult brain and during brain aging. Neuron. 2010;68:409–27. 21040844 10.1016/j.neuron.2010.09.043 PMC3056408

[19] Elahy M, Jackaman C, Mamo JC, Lam V, Dhaliwal SS, Giles C, et al. Blood-brain barrier dysfunction developed during normal aging is associated with inflammation and loss of tight junctions but not with leukocyte recruitment. Immun Ageing. 2015;12:2. 25784952 10.1186/s12979-015-0029-9 PMC4362825

[20] Soto I, Graham LC, Richter HJ, Simeone SN, Radell JE, Grabowska W, et al. APOE stabilization by exercise prevents aging neurovascular dysfunction and complement induction. PLoS Biol. 2015;13:e1002279. 26512759 10.1371/journal.pbio.1002279 PMC4626092

[21] Yang AC, Stevens MY, Chen MB, Lee DP, Stahli D, Gate D, et al. Physiological blood-brain transport is impaired with age by a shift in transcytosis. Nature. 2020;583:425–30. 32612231 10.1038/s41586-020-2453-z PMC8331074

[22] Brown AL, Smith DW. Improved RNA preservation for immunolabeling and laser microdissection. RNA. 2009;15:2364–74. 19850907 10.1261/rna.1733509 PMC2779672

[23] Tsumagari K, Sato Y, Aoyagi H, Okano H, Kuromitsu J. Proteomic characterization of aging-driven changes in the mouse brain by co-expression network analysis. Sci Rep. 2023;13:18191. 37875604 10.1038/s41598-023-45570-w PMC10598061

[24] Todorov-Volgyi K, Gonzalez-Gallego J, Muller SA, Beaufort N, Malik R, Schifferer M, et al. Proteomics of mouse brain endothelium uncovers dysregulation of vesicular transport pathways during aging. Nat Aging. 2024;4:595–612. 38519806 10.1038/s43587-024-00598-z

[25] Vanlandewijck M, He L, Mae MA, Andrae J, Ando K, Del Gaudio F, et al. A molecular atlas of cell types and zonation in the brain vasculature. Nature. 2018;554:475–80. 29443965 10.1038/nature25739

[26] Kalucka J, de Rooij L, Goveia J, Rohlenova K, Dumas SJ, Meta E, et al. Single-cell transcriptome atlas of murine endothelial cells. Cell. 2020;180(764–779):e720. 10.1016/j.cell.2020.01.015 32059779

[27] Ashby JW, Mack JJ. Endothelial control of cerebral blood flow. Am J Pathol. 2021;191:1906–16. 33713686 10.1016/j.ajpath.2021.02.023

[28] Hall CN, Reynell C, Gesslein B, Hamilton NB, Mishra A, Sutherland BA, et al. Capillary pericytes regulate cerebral blood flow in health and disease. Nature. 2014;508:55–60. 24670647 10.1038/nature13165 PMC3976267

[29] Zlokovic BV. The blood-brain barrier in health and chronic neurodegenerative disorders. Neuron. 2008;57:178–201. 18215617 10.1016/j.neuron.2008.01.003

[30] Munji RN, Soung AL, Weiner GA, Sohet F, Semple BD, Trivedi A, et al. Profiling the mouse brain endothelial transcriptome in health and disease models reveals a core blood-brain barrier dysfunction module. Nat Neurosci. 2019;22:1892–902. 31611708 10.1038/s41593-019-0497-x PMC6858546

[31] Satija R, Farrell JA, Gennert D, Schier AF, Regev A. Spatial reconstruction of single-cell gene expression data. Nat Biotechnol. 2015;33:495–502. 25867923 10.1038/nbt.3192 PMC4430369

[32] Zhan R, Meng X, Tian D, Xu J, Cui H, Yang J, et al. NAD(+) rescues aging-induced blood-brain barrier damage via the CX43-PARP1 axis. Neuron. 2023;111(3634–3649):e3637. 10.1016/j.neuron.2023.08.010 37683629

[33] Kim J, Cooper JA. Junctional localization of septin 2 is required for organization of junctional proteins in static endothelial monolayers. Arterioscler Thromb Vasc Biol. 2021;41:346–59. 33147991 10.1161/ATVBAHA.120.315472 PMC7769918

[34] Xu S, Xu Y, Liu P, Zhang S, Liu H, Slavin S, et al. The novel coronary artery disease risk gene JCAD/KIAA1462 promotes endothelial dysfunction and atherosclerosis. Eur Heart J. 2019;40:2398–408. 31539914 10.1093/eurheartj/ehz303 PMC6698662

[35] Douglas G, Mehta V, Al Haj Zen A, Akoumianakis I, Goel A, Rashbrook VS, et al. A key role for the novel coronary artery disease gene JCAD in atherosclerosis via shear stress mechanotransduction. Cardiovasc Res. 2020;116:1863–74. 31584065 10.1093/cvr/cvz263 PMC7449560

[36] Sweeney MD, Zhao Z, Montagne A, Nelson AR, Zlokovic BV. Blood-brain barrier: from physiology to disease and back. Physiol Rev. 2019;99:21–78. 30280653 10.1152/physrev.00050.2017 PMC6335099

[37] Hashimoto Y, Greene C, Munnich A, Campbell M. The CLDN5 gene at the blood-brain barrier in health and disease. Fluids Barriers CNS. 2023;20:22. 36978081 10.1186/s12987-023-00424-5 PMC10044825

[38] Mandel I, Paperna T, Volkowich A, Merhav M, Glass-Marmor L, Miller A. The ubiquitin-proteasome pathway regulates claudin 5 degradation. J Cell Biochem. 2012;113:2415–23. 22389112 10.1002/jcb.24118

[39] Ventura-Antunes A, Herculano-Houzel S. Energy supply per neuron is constrained by capillary density in the mouse brain. Front Integr Neurosci. 2022;16:760887. 36105258 10.3389/fnint.2022.760887 PMC9465999

[40] Winkler EA, Sengillo JD, Bell RD, Wang J, Zlokovic BV. Blood-spinal cord barrier pericyte reductions contribute to increased capillary permeability. J Cereb Blood Flow Metab. 2012;32:1841–52. 22850407 10.1038/jcbfm.2012.113 PMC3463878

[41] Wang Y, Taylor E, Zikopoulos B, Seta F, Huang N, Hamilton JA, et al. Aging-induced microbleeds of the mouse thalamus compared to sensorimotor and memory defects. Neurobiol Aging. 2021;100:39–47. 33477010 10.1016/j.neurobiolaging.2020.11.017 PMC8162167

[42] Taylor EN, Huang N, Wisco J, Wang Y, Morgan KG, Hamilton JA. The brains of aged mice are characterized by altered tissue diffusion properties and cerebral microbleeds. J Transl Med. 2020;18:277. 32641073 10.1186/s12967-020-02441-6 PMC7346388

[43] Gordon BA, Blazey T, Benzinger TL, Head D. Effects of aging and Alzheimer’s disease along the longitudinal axis of the hippocampus. J Alzheimers Dis. 2013;37:41–50. 23780659 10.3233/JAD-130011 PMC3883500

[44] Bettio LEB, Rajendran L, Gil-Mohapel J. The effects of aging in the hippocampus and cognitive decline. Neurosci Biobehav Rev. 2017;79:66–86. 28476525 10.1016/j.neubiorev.2017.04.030

[45] O’Shea A, Cohen RA, Porges EC, Nissim NR, Woods AJ. Cognitive aging and the hippocampus in older adults. Front Aging Neurosci. 2016;8:298. 28008314 10.3389/fnagi.2016.00298 PMC5143675

[46] Nation DA, Sweeney MD, Montagne A, Sagare AP, D’Orazio LM, Pachicano M, et al. Blood-brain barrier breakdown is an early biomarker of human cognitive dysfunction. Nat Med. 2019;25:270–6. 30643288 10.1038/s41591-018-0297-y PMC6367058

[47] Mooradian AD, Haas MJ, Chehade JM. Age-related changes in rat cerebral occludin and zonula occludens-1 (ZO-1). Mech Ageing Dev. 2003;124:143–6. 12633933 10.1016/s0047-6374(02)00041-6

[48] Murugesan N, Demarest TG, Madri JA, Pachter JS. Brain regional angiogenic potential at the neurovascular unit during normal aging. Neurobiol Aging. 2012;33(1004):e1001-1016. 10.1016/j.neurobiolaging.2011.09.022 PMC3266473 22019053

[49] Happonen KE, Furst CM, Saxne T, Heinegard D, Blom AM. PRELP protein inhibits the formation of the complement membrane attack complex. J Biol Chem. 2012;287:8092–100. 22267731 10.1074/jbc.M111.291476 PMC3318720

[50] Nikolakopoulou AM, Montagne A, Kisler K, Dai Z, Wang Y, Huuskonen MT, et al. Pericyte loss leads to circulatory failure and pleiotrophin depletion causing neuron loss. Nat Neurosci. 2019;22:1089–98. 31235908 10.1038/s41593-019-0434-z PMC6668719

[51] Krellman JW, Ruiz HH, Marciano VA, Mondrow B, Croll SD. Behavioral and neuroanatomical abnormalities in pleiotrophin knockout mice. PLoS ONE. 2014;9:e100597. 25000129 10.1371/journal.pone.0100597 PMC4085064

[52] Abadier M, Haghayegh Jahromi N, Cardoso Alves L, Boscacci R, Vestweber D, Barnum S, et al. Cell surface levels of endothelial ICAM-1 influence the transcellular or paracellular T-cell diapedesis across the blood-brain barrier. Eur J Immunol. 2015;45:1043–58. 25545837 10.1002/eji.201445125

[53] Ximerakis M, Lipnick SL, Innes BT, Simmons SK, Adiconis X, Dionne D, et al. Single-cell transcriptomic profiling of the aging mouse brain. Nat Neurosci. 2019;22:1696–708. 31551601 10.1038/s41593-019-0491-3

[54] Propson NE, Roy ER, Litvinchuk A, Kohl J, Zheng H. Endothelial C3a receptor mediates vascular inflammation and blood-brain barrier permeability during aging. J Clin Invest. 2021;131. 10.1172/JCI140966 PMC7773352 32990682

[55] Lopez-Otin C, Blasco MA, Partridge L, Serrano M, Kroemer G. Hallmarks of aging: an expanding universe. Cell. 2023;186:243–78. 36599349 10.1016/j.cell.2022.11.001

[56] Reed MJ, Vernon RB, Damodarasamy M, Chan CK, Wight TN, Bentov I, et al. Microvasculature of the mouse cerebral cortex exhibits increased accumulation and synthesis of hyaluronan with aging. J Gerontol A Biol Sci Med Sci. 2017;72:740–6. 28482035 10.1093/gerona/glw213 PMC6075594

[57] Bennett HC, Zhang Q, Wu YT, Manjila SB, Chon U, Shin D, et al. Aging drives cerebrovascular network remodeling and functional changes in the mouse brain. Nat Commun. 2024;15:6398. 39080289 10.1038/s41467-024-50559-8 PMC11289283

[58] Ceafalan LC, Fertig TE, Gheorghe TC, Hinescu ME, Popescu BO, Pahnke J, et al. Age-related ultrastructural changes of the basement membrane in the mouse blood-brain barrier. J Cell Mol Med. 2019;23:819–27. 30450815 10.1111/jcmm.13980 PMC6349169

[59] Frias-Anaya E, Gromnicova R, Kraev I, Rogachevsky V, Male DK, Crea F, et al. Age-related ultrastructural neurovascular changes in the female mouse cortex and hippocampus. Neurobiol Aging. 2021;101:273–84. 33579556 10.1016/j.neurobiolaging.2020.12.008

[60] Armulik A, Genove G, Mae M, Nisancioglu MH, Wallgard E, Niaudet C, et al. Pericytes regulate the blood-brain barrier. Nature. 2010;468:557–61. 20944627 10.1038/nature09522

[61] Sykova E, Mazel T, Hasenohrl RU, Harvey AR, Simonova Z, Mulders WH, et al. Learning deficits in aged rats related to decrease in extracellular volume and loss of diffusion anisotropy in hippocampus. Hippocampus. 2002;12:269–79. 12000123 10.1002/hipo.1101

[62] Nitta T, Hata M, Gotoh S, Seo Y, Sasaki H, Hashimoto N, et al. Size-selective loosening of the blood-brain barrier in claudin-5-deficient mice. J Cell Biol. 2003;161:653–60. 12743111 10.1083/jcb.200302070 PMC2172943

[63] Noorani B, Chowdhury EA, Alqahtani F, Ahn Y, Nozohouri E, Zoubi S, et al. Effects of volatile anesthetics versus ketamine on blood-brain barrier permeability via lipid-mediated alterations of endothelial cell membranes. J Pharmacol Exp Ther. 2023;385:135–45. 36828631 10.1124/jpet.122.001281

[64] Cao Y, Ni C, Li Z, Li L, Liu Y, Wang C, et al. Isoflurane anesthesia results in reversible ultrastructure and occludin tight junction protein expression changes in hippocampal blood-brain barrier in aged rats. Neurosci Lett. 2015;587:51–6. 25524410 10.1016/j.neulet.2014.12.018

[65] Rizk AA, Plitman E, Senthil P, Venkatraghavan L, Chowdhury T. Effects of anesthetic agents on blood brain barrier integrity: a systematic review. Can J Neurol Sci. 2023;50:897–904. 36353901 10.1017/cjn.2022.319

[66] Kim H, Noh M, Zhang H, Kim Y, Park S, Park J, et al. Long-term administration of CU06-1004 ameliorates cerebrovascular aging and BBB injury in aging mouse model. Fluids Barriers CNS. 2023;20:9. 36726154 10.1186/s12987-023-00410-x PMC9893613

[67] Takechi R, Pallebage-Gamarallage MM, Lam V, Giles C, Mamo JC. Aging-related changes in blood-brain barrier integrity and the effect of dietary fat. Neurodegener Dis. 2013;12:125–35. 23128303 10.1159/000343211

[68] Wekerle H. Brain autoimmunity and intestinal microbiota: 100 trillion game changers. Trends Immunol. 2017;38:483–97. 28601415 10.1016/j.it.2017.03.008

[69] Kadowaki A, Quintana FJ. The gut-CNS axis in multiple sclerosis. Trends Neurosci. 2020;43:622–34. 32650957 10.1016/j.tins.2020.06.002 PMC8284847

[70] Erny D, Hrabe de Angelis AL, Jaitin D, Wieghofer P, Staszewski O, David E, et al. Host microbiota constantly control maturation and function of microglia in the CNS. Nat Neurosci. 2015;18:965–77. 26030851 10.1038/nn.4030 PMC5528863

[71] Wu Y, Li P, Bhat N, Fan H, Liu M. Effects of repeated sleep deprivation on brain pericytes in mice. Sci Rep. 2023;13:12760. 37550395 10.1038/s41598-023-40138-0 PMC10406921

[72] Yoshino J, Baur JA, Imai SI. NAD(+) intermediates: the biology and therapeutic potential of NMN and NR. Cell Metab. 2018;27:513–28. 29249689 10.1016/j.cmet.2017.11.002 PMC5842119

[73] Shao X, Shou Q, Felix K, Ojogho B, Jiang X, Gold BT, et al. Age-related decline in blood-brain barrier function is more pronounced in males than females in parietal and temporal regions. bioRxiv. 2024. 10.7554/eLife.96155 PMC11534331 39495221

[74] Toth P, Tarantini S, Springo Z, Tucsek Z, Gautam T, Giles CB, et al. Aging exacerbates hypertension-induced cerebral microhemorrhages in mice: role of resveratrol treatment in vasoprotection. Aging Cell. 2015;14:400–8. 25677910 10.1111/acel.12315 PMC4406669

[75] Sumbria RK, Grigoryan MM, Vasilevko V, Paganini-Hill A, Kilday K, Kim R, et al. Aging exacerbates development of cerebral microbleeds in a mouse model. J Neuroinflammation. 2018;15:69. 29510725 10.1186/s12974-018-1092-x PMC5840821

