# Supplementary material for: Aging disrupts blood–brain and blood-spinal cord barrier homeostasis, but does not increase paracellular permeability
Source: GeroScience. 2024 Oct 30;47(1):263–85. doi: 10.1007/s11357-024-01404-9 (PMC11872845; doi:10.1007/s11357-024-01404-9)
Supplement: Supplementary file 6 — Supplementary file6 (DOCX 19397 KB) [file 11357_2024_1404_MOESM6_ESM.docx]

Supplementary Figures

**Fig. S1** Western blotting in aging mouse cortex **(a)** normalised fluorescence of Cldn5 in young (red) and old (blue). Cldn5 fluorescence was normalised to Tubb3 and revert total protein fluorescence. Points are individual animals. Bars are mean and error bars ±SD. **(b)** Cldn5 Western blot. **(c)** Cldn5 revert total protein control. **(d)** normalised fluorescence of Ocln in young (red) and old (blue). Ocln fluorescence was normalised to Tubb3 and revert total protein fluorescence. Points are individual animals. Bars are mean and error bars ±SD. **(e)** Ocln Western blot. **(f)** Ocln revert total protein control. **(g)** normalised fluorescence of Tubb3 in young (red) and old (blue). Tubb3 fluorescence was normalised to revert total protein fluorescence. Points are individual animals. Bars are mean and error bars ±SD. **(h)** Tubb3 Western blot. **(i)** Tubb3 revert total protein control. Y – young ; O - old


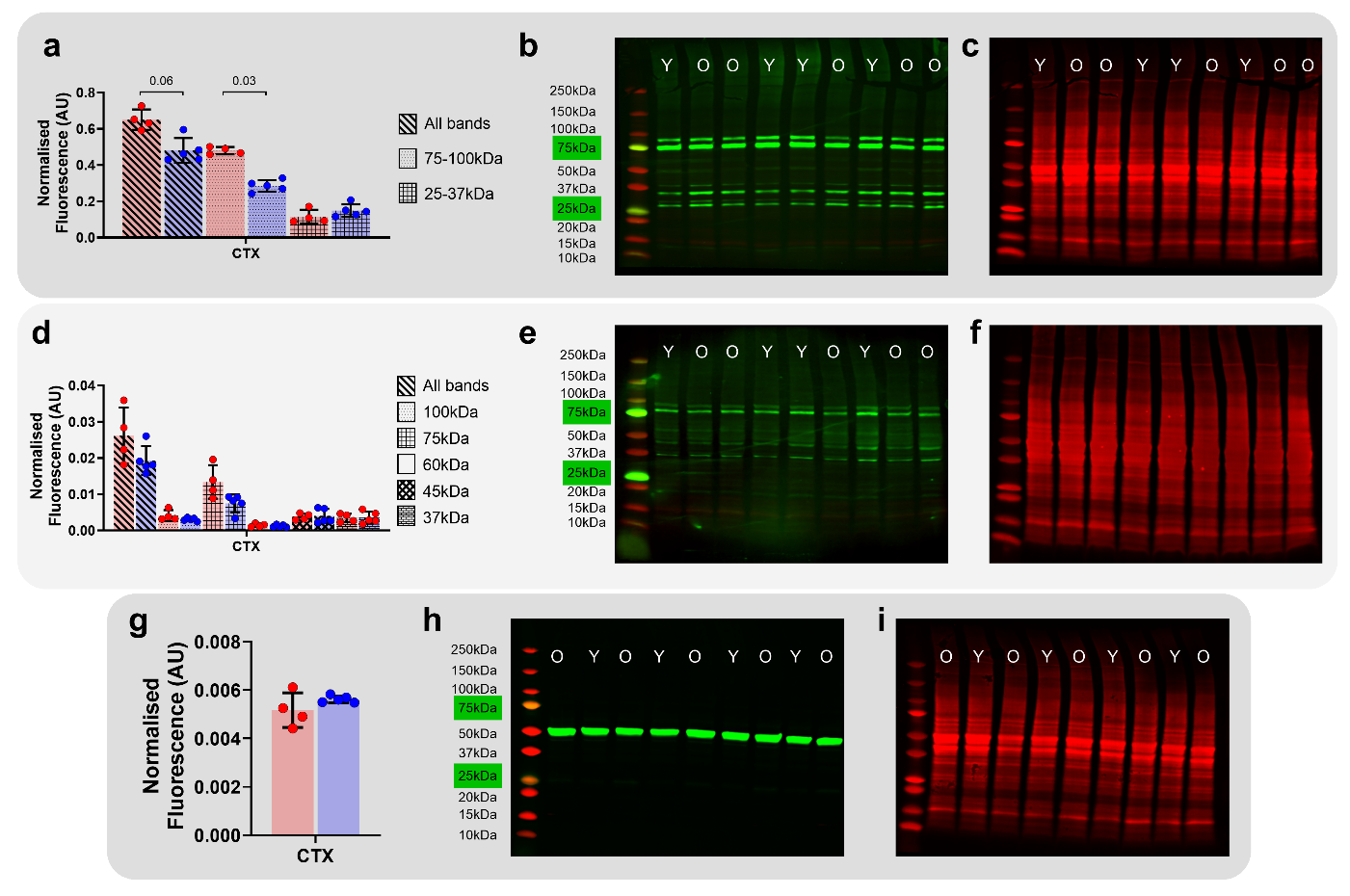


**Fig. S2** Western blotting in aging mouse spinal cord **(a)** normalised fluorescence of Cldn5 in young (red) and old (blue). Cldn5 fluorescence was normalised to Tubb3 and revert total protein fluorescence. Points are individual animals. Bars are mean and error bars ±SD. **(b)** Cldn5 Western blot. **(c)** Cldn5 revert total protein control. **(d)** normalised fluorescence of Ocln in young (red) and old (blue). Ocln fluorescence was normalised to Tubb3 and revert total protein fluorescence. Points are individual animals. Bars are mean and error bars ±SD. **(e)** Ocln Western blot. **(f)** Ocln revert total protein control. **(g)** normalised fluorescence of Tubb3 in young (red) and old (blue). Tubb3 fluorescence was normalised to revert total protein fluorescence. Points are individual animals. Bars are mean and error bars ±SD. **(h)** Tubb3 Western blot. **(i)** Tubb3 revert total protein control. Y – young ; O - old


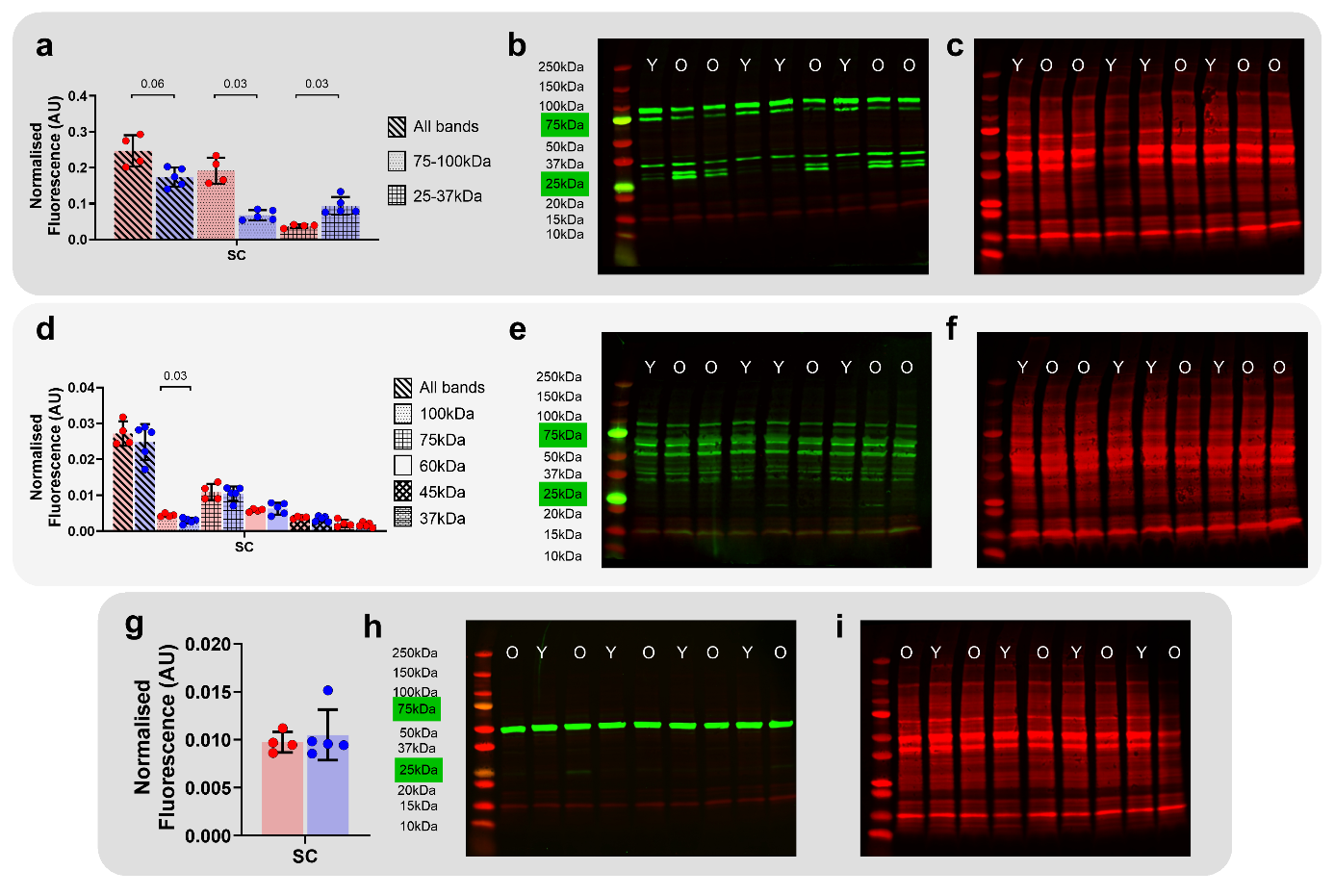


**Fig. S3** Wet and dry weight of the aging **(a)** brain and **(b)** spinal cord. Points are individual animals. Bars are mean and error bars ±SD. ** p-value <0.01


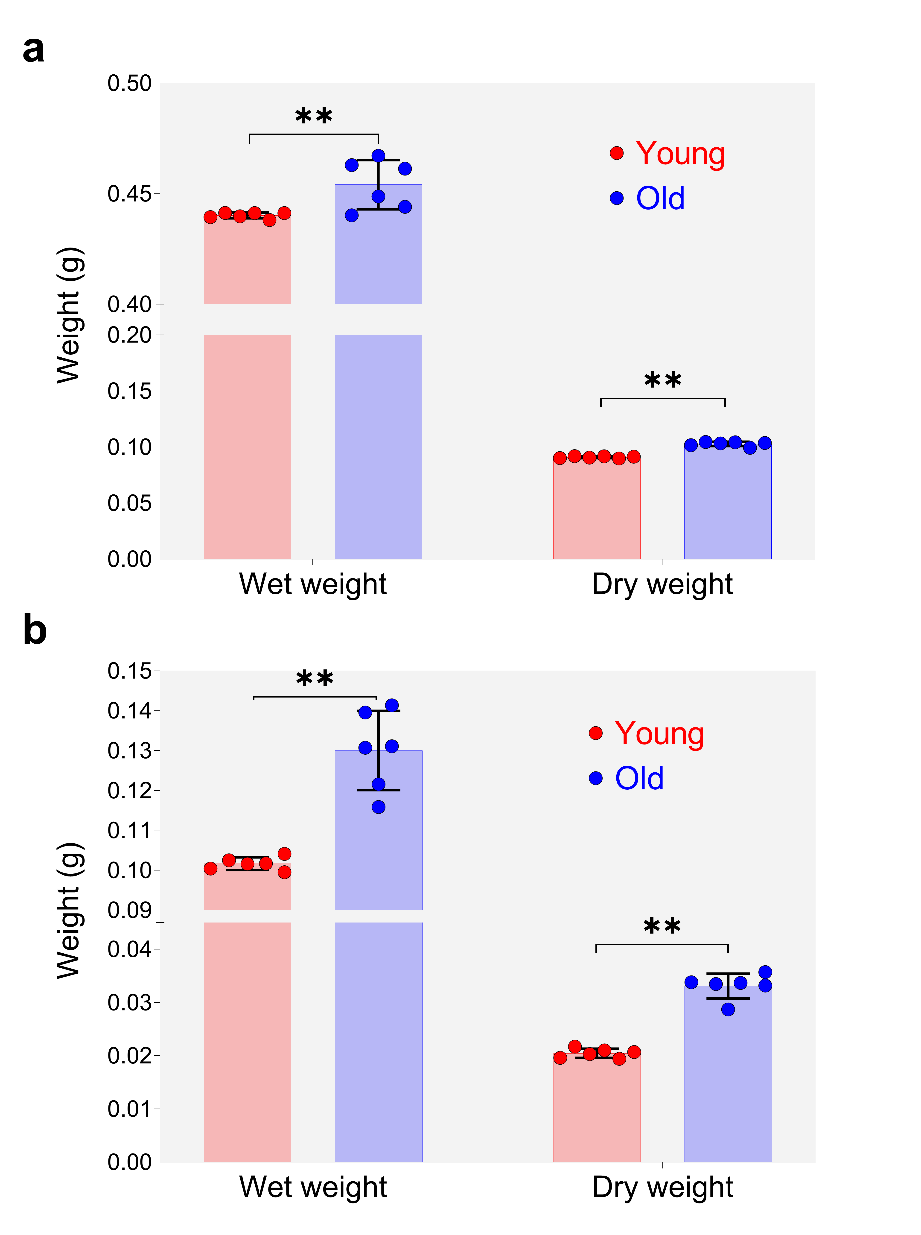


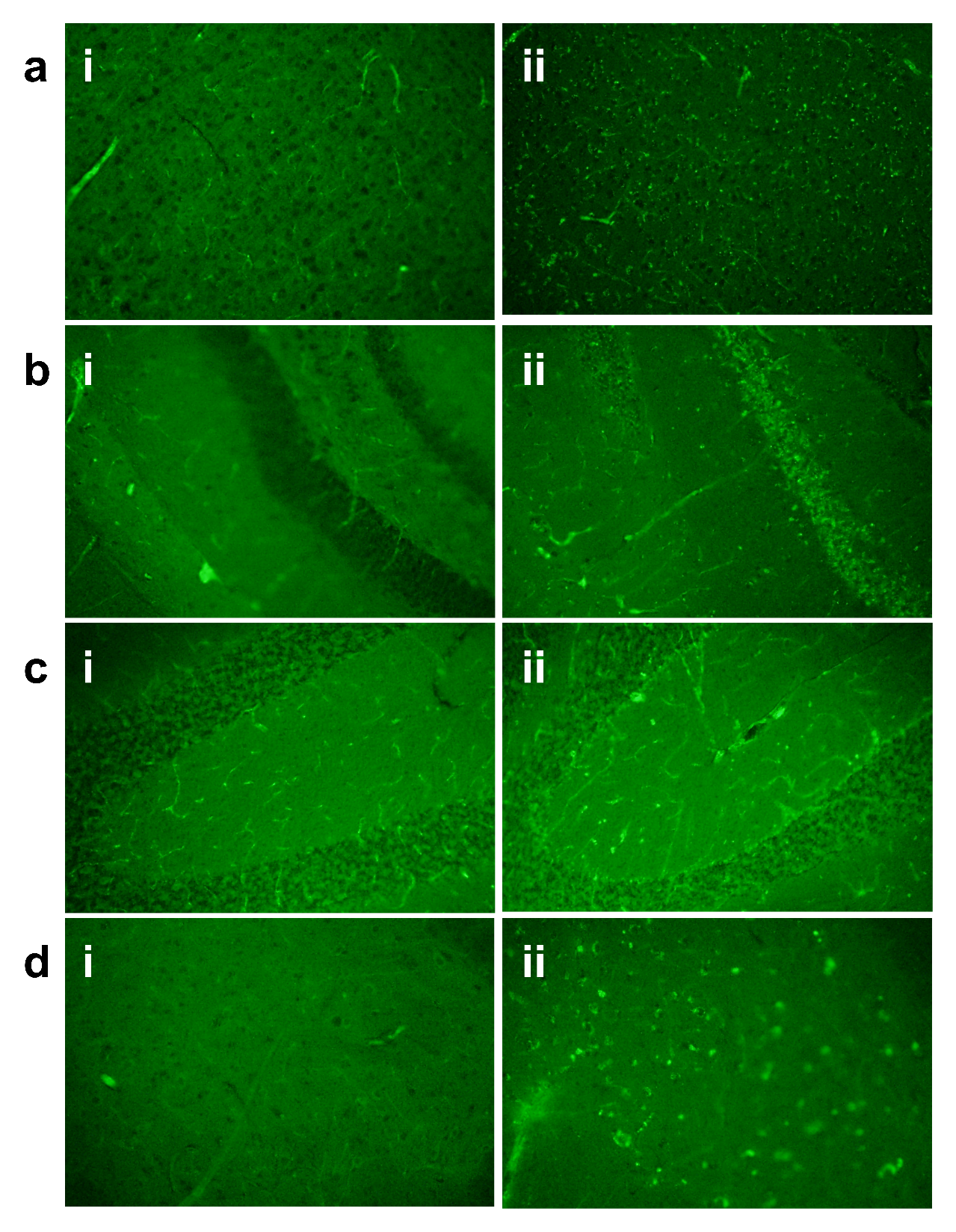


**Fig. S4** Dextran in the aging CNS. Example image of dextran distribution in the **(a)** cortex, **(b)** hippocampus, **(c)** cerebellum, **(d)** spinal cord in **(i)** young and **(ii)** old. 20x


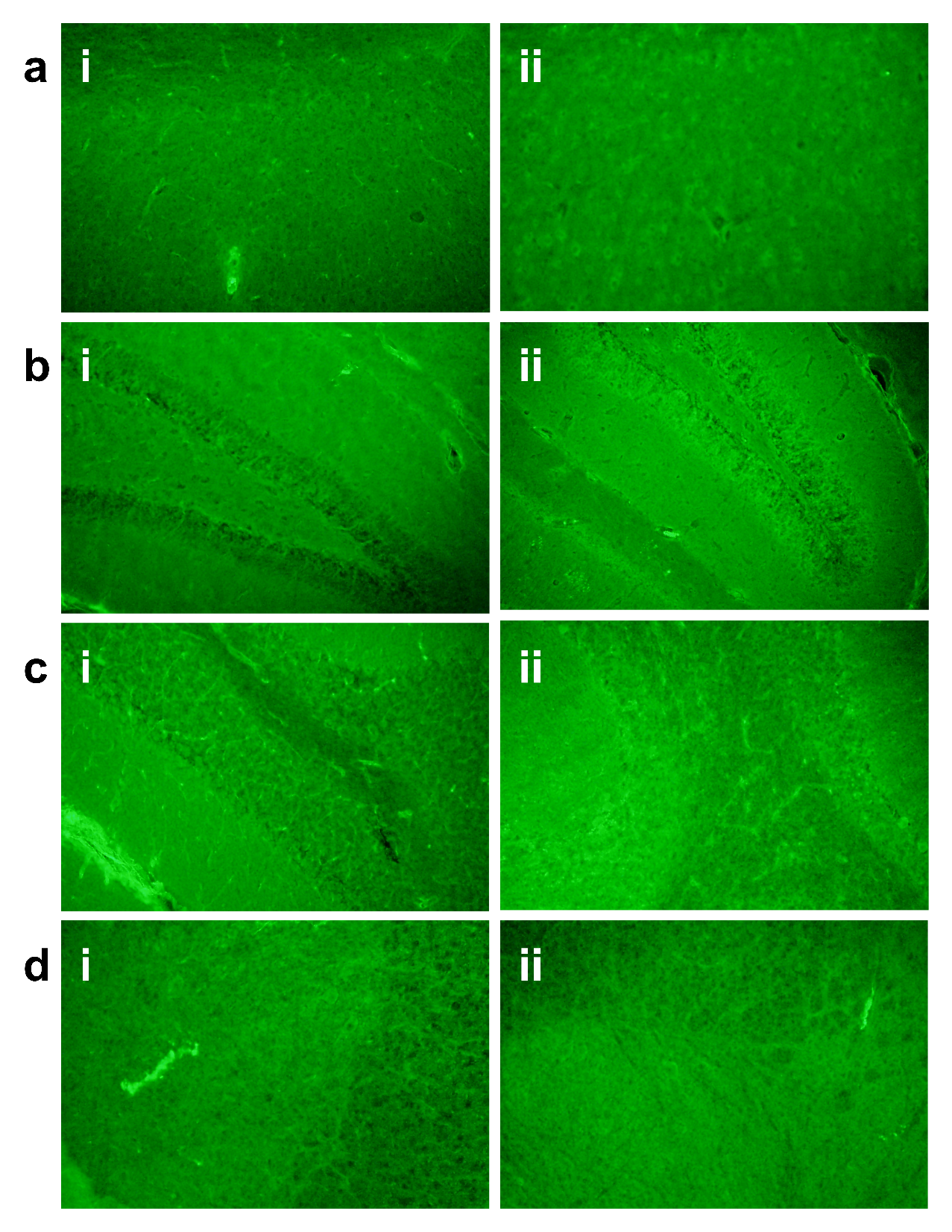


**Fig. S5** Serum albumin labelling in the aging CNS. Example image of serum albumin labelling in the **(a)** cortex, **(b)** hippocampus, **(c)** cerebellum, **(d)** spinal cord in **(i)** young and **(ii)** old. 20x


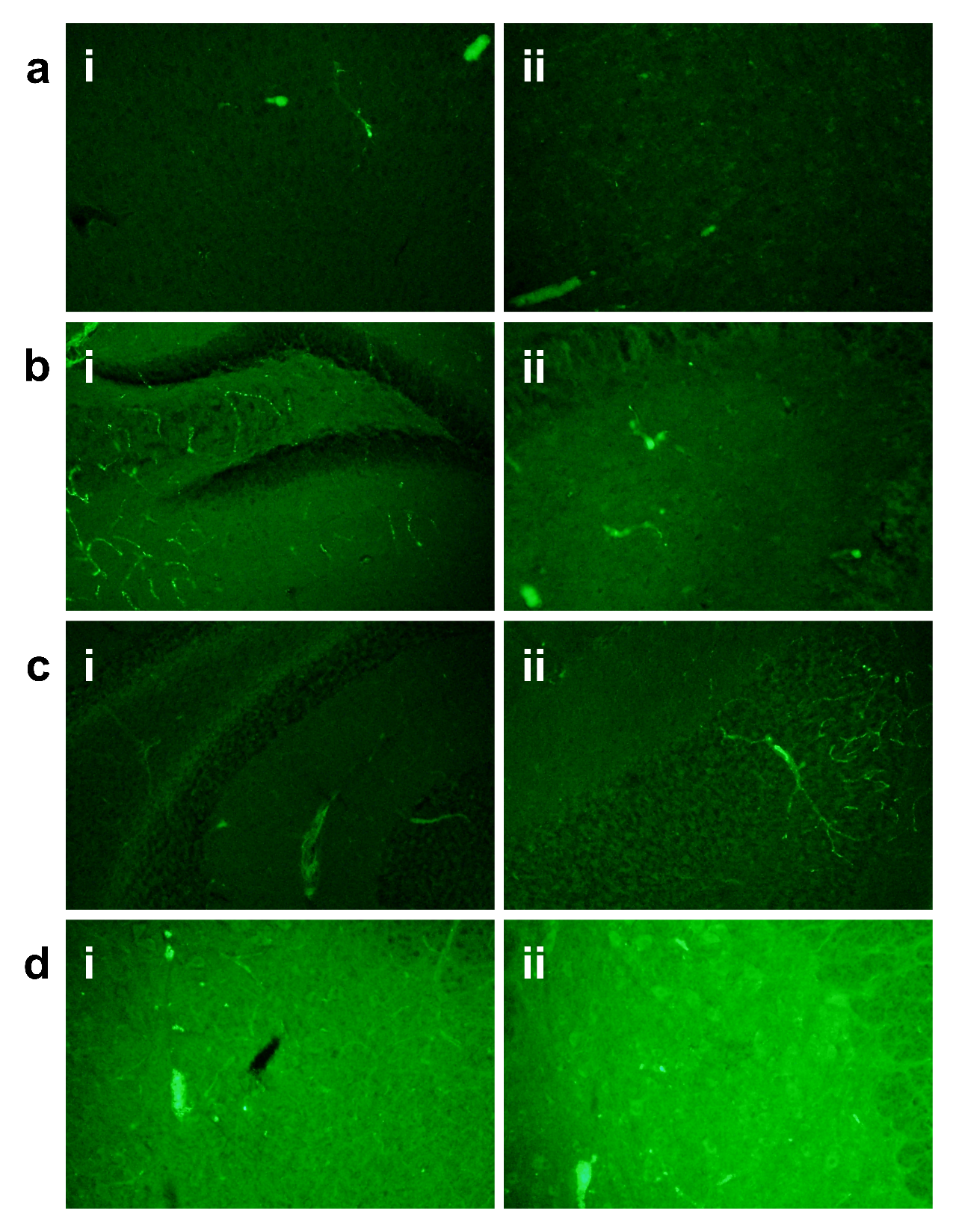


**Fig. S6** IgG labelling in the aging CNS. Example image of IgG labelling in the **(a)** cortex, **(b)** hippocampus, **(c)** cerebellum, **(d)** spinal cord in **(i)** young and **(ii)** old. 20x


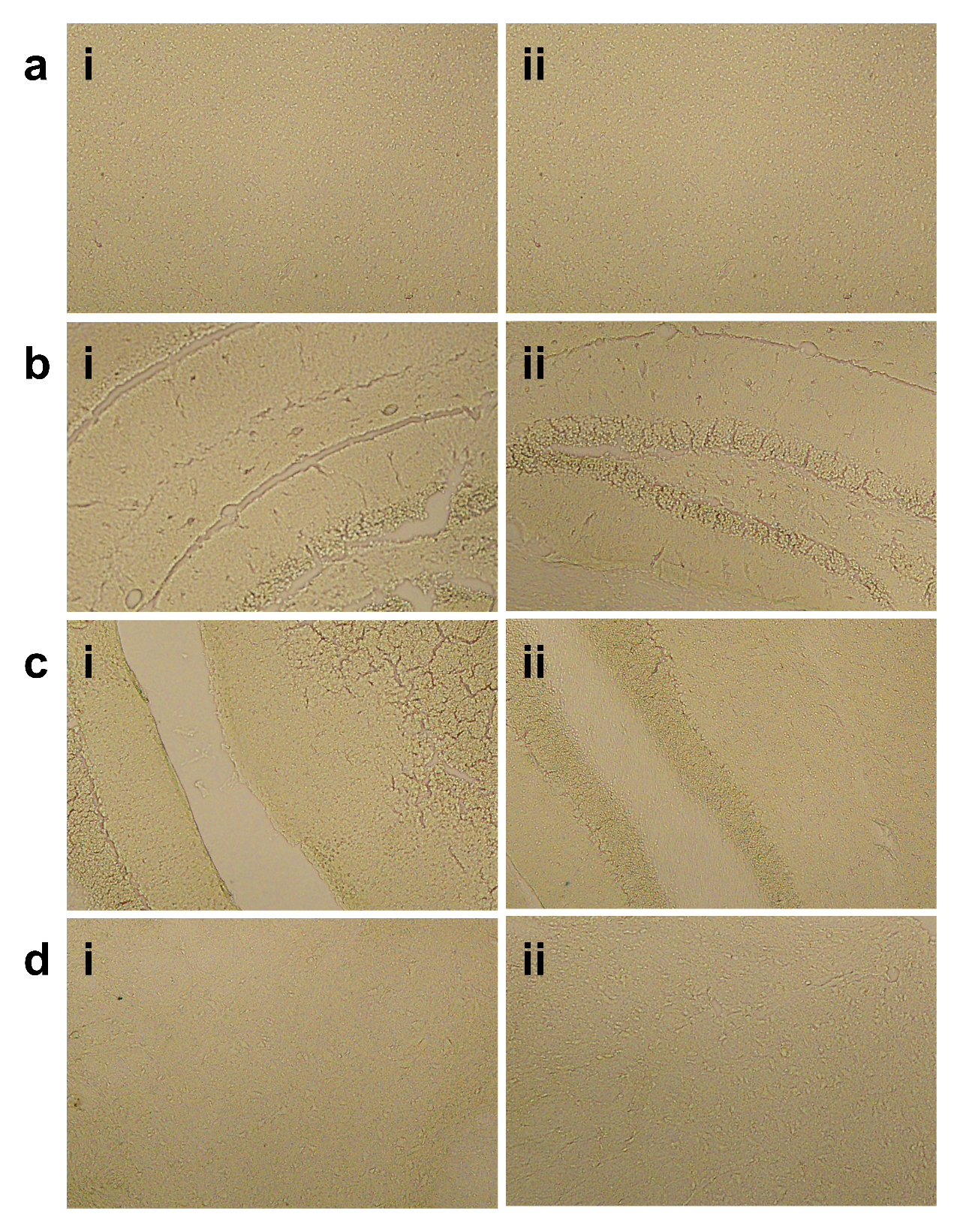


**Fig. S7** Iron labelling in the aging CNS. Example image of iron labelling in the **(a)** cortex, **(b)** hippocampus, **(c)** cerebellum, **(d)** spinal cord in **(i)** young and **(ii)** old. 20x


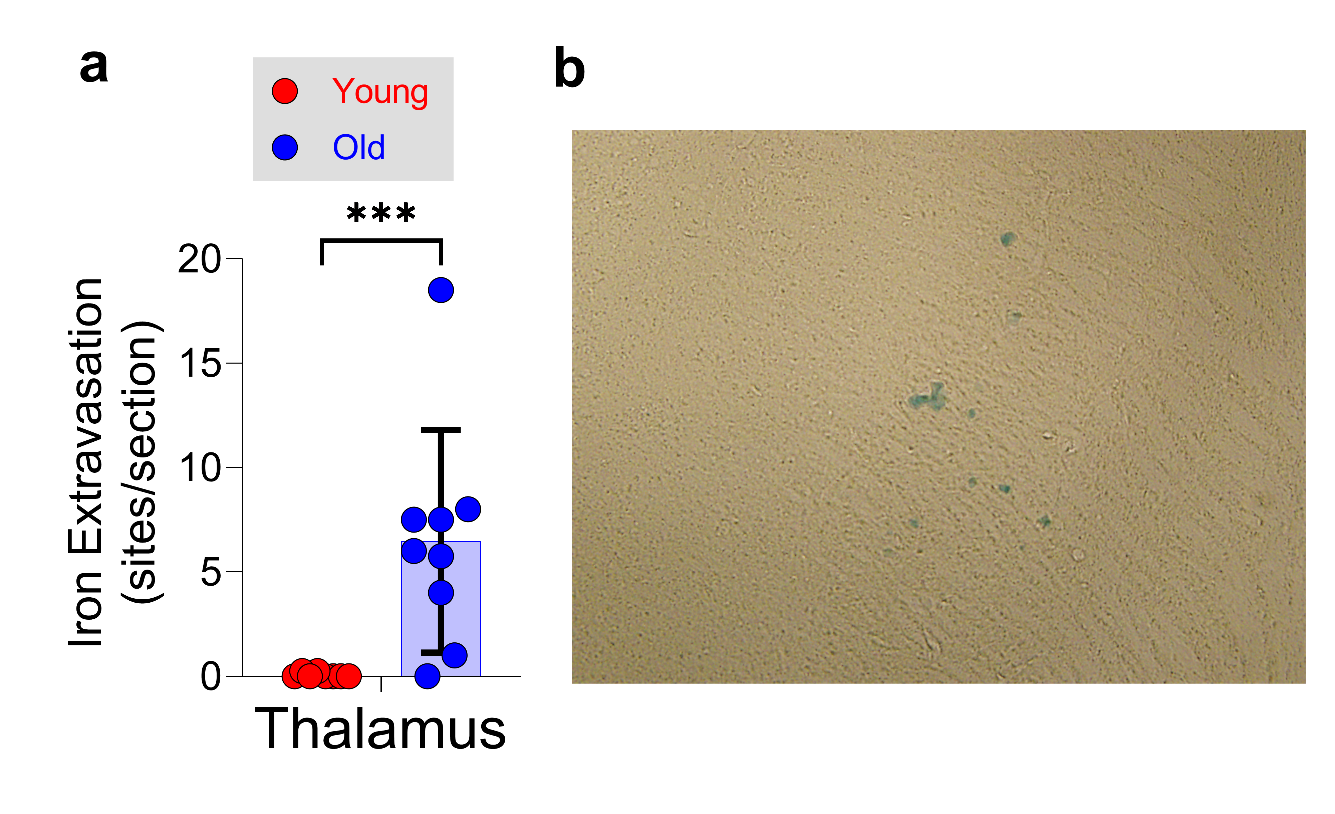


**Fig. S8** Iron labelling in the aging thalamus. **(a)** Number of sites per section of iron extravasation in young and old thalamus (n=8/9/grp). Points are individual animals. *** p-value <0.001. **(b)** Example image of thalamic iron labelling. 10x


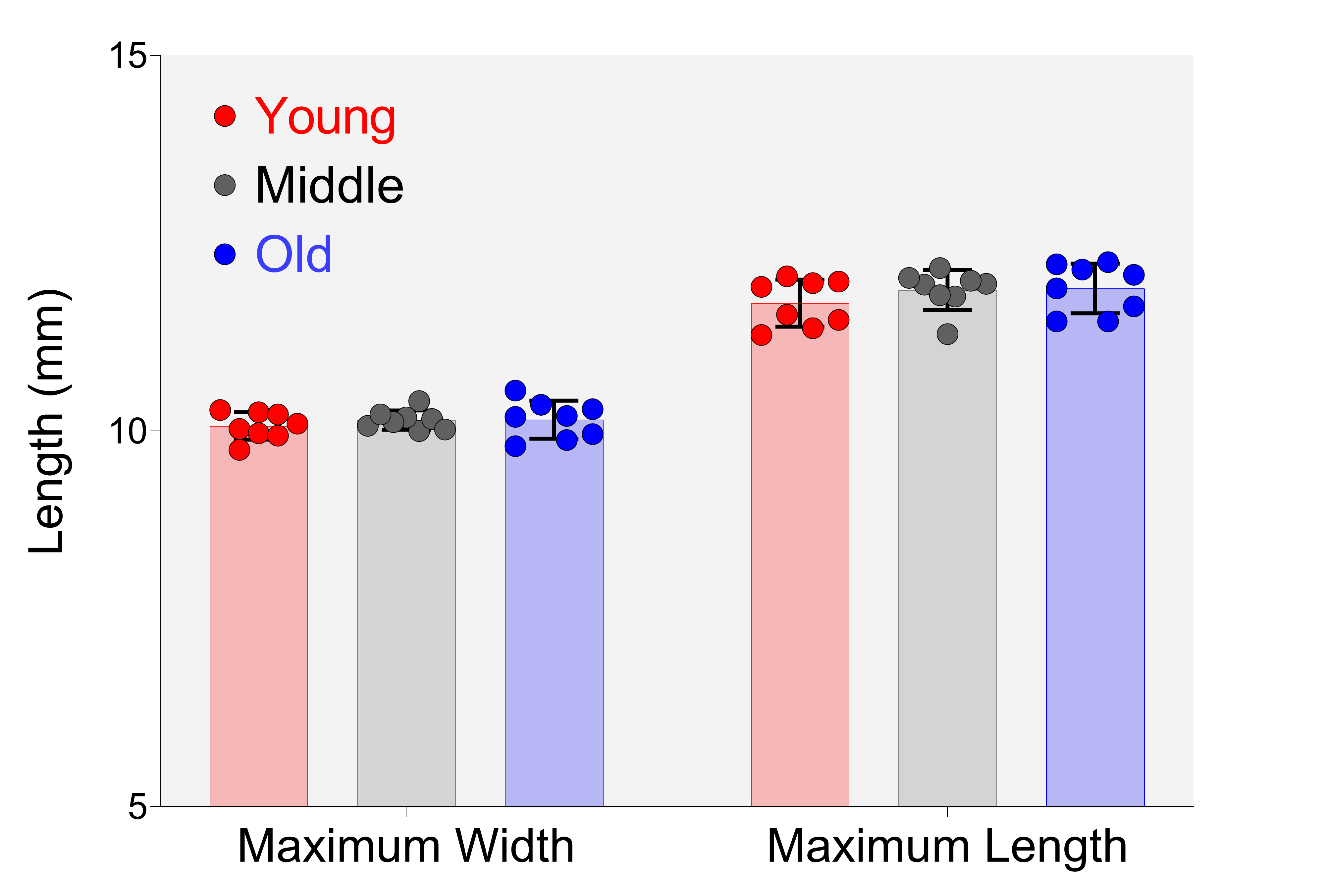


**Fig. S9** Gross measurements of the aging brain. Maximum width and length of the brains from young, middle-age, and old animal (n=8/gp) were measured using calipers. Points are individual animals. Bars are mean and error bars ±SD. No comparisons were significant by Kruskal-Wallis Test
